# Exercise-induced irisin ameliorates cognitive impairment following chronic cerebral hypoperfusion by suppressing neuroinflammation and hippocampal neuronal apoptosis

**DOI:** 10.1186/s12974-025-03493-5

**Published:** 2025-06-28

**Authors:** Weiping Xiao, Yibing Yang, Lu Bai, Peixuan Yang, Runze Li, Daizhi Yang, Fanying Li, Lingzhi Quan, Qiupeng Liang, Yan Yan, Tiewei Qi, Feng Liang

**Affiliations:** 1 Department of Neurosurgery, The First Affiliated Hospital of Sun Yat-sen University, Guangzhou, Guangdong People’s Republic of China; 2 Department of Rehabilitation Medicine, The First Affiliated Hospital of Sun Yat-sen University, Guangzhou, Guangdong People’s Republic of China; 3 Department of Endocrinology and Metabolism, Guangdong Provincial Key Laboratory of Diabetology, The Third Affiliated Hospital of Sun Yat-sen University, Guangzhou, Guangdong People’s Republic of China

**Keywords:** Chronic cerebral hypoperfusion, Irisin, Aerobic exercise, Neuronal apoptosis, Neuroinflammation

## Abstract

**Background:**

Chronic cerebral hypoperfusion (CCH) is a pathophysiological hallmark of vascular dementia, the second most common form of dementia. CCH exerts complex and subtle detrimental effects on both the brain and peripheral systems. Irisin is a polypeptide primarily expressed in contracting skeletal muscle and the brain. However, its role in CCH remains unclear. This study aimed to investigate the effects of CCH on irisin metabolism and whether increasing endogenous irisin levels through forced aerobic exercise (FAE) could confer neuroprotection against secondary brain injury induced by CCH.

**Methods:**

A total of 212 adult (8-week-old) male C57BL/6 mice were randomly assigned to either sham or CCH groups. CCH was induced by bilateral common carotid artery stenosis. FAE consisted of daily swimming (1 h/day, 5 days/week, for 5 weeks). Two subgroups of CCH mice received daily intraperitoneal injections of either DMSO or cilengitide trifluoroacetate (CT), a selective inhibitor of integrin αV and β5 (the irisin receptor), during FAE. ELISA and western blotting were used to assess irisin expression, while western blotting, TUNEL, immunofluorescence staining, and neurobehavioral tests were conducted to evaluate neurofunctional outcomes.

**Results:**

Hippocampal and serum irisin levels were progressively reduced in CCH mice. Additionally, expression of integrins αV and β5 in hippocampal neurons, microglia, and astrocytes decreased post-CCH. FAE effectively enhanced both peripheral and central irisin expression. Increased endogenous irisin levels inhibited CCH-induced hippocampal neuronal apoptosis and microglial activation, thereby promoting neuronal survival and partially ameliorating white matter injury. These changes led to improvements in memory, motor function, and anxiety- and depression-like behaviors. Mechanistically, the neuroprotective effects of irisin were mediated by enhanced hippocampal neuronal and microglial autophagy through increased AMPK phosphorylation and decreased mTOR phosphorylation—effects abolished by CT treatment.

**Conclusion:**

Our findings demonstrate that enhancing endogenous irisin via FAE mitigates CCH-induced neuronal apoptosis, microglial activation, cognitive impairment, and affective behavioral deficits by promoting autophagy through the integrin αVβ5/AMPK/mTOR signaling pathway.

**Supplementary Information:**

The online version contains supplementary material available at 10.1186/s12974-025-03493-5.

## Introduction

Vascular dementia (VaD) is the second most prevalent form of dementia caused by cerebrovascular diseases. It is estimated that VaD accounts for approximately 15–20% of dementia cases in North America and Europe, and up to 30% in Asia [1–4]. Cognitive processes include acquisition, comprehension, and retention of knowledge through sensation, experience, and thought. Brain function critically depends on sustained supply of oxygen and glucose to neurons, as well as efficient removal of various metabolic byproducts, to meet the demands of neuronal activity. Cerebrovascular diseases damage specific brain areas, such as frontal and temporal lobes, and the limbic system, and can lead to cognitive impairments, depressive-like behaviors, and even the most severe stage as VaD. In addition, metabolic diseases including hyperlipidemia, hypertension, and diabetes can impair autoregulation of cerebral microvasculature. Moreover, certain clinical conditions, including chronic heart failure, atherosclerosis of large and small vessels, and carotid artery stenosis, can ultimately lead to reduced cerebral blood flow and chronic cerebral hypoperfusion (CCH) [5]. CCH has been considered as a pathophysiological hallmark of VaD [6]. The aging population is bound to increase the incidence of above-mentioned diseases and VaD, thereby imposing a heavy burden on social and healthcare systems. Since no effective treatment for VaD has been found, in addition to seeking therapeutic methods for the related diseases, it is imperative to explore from the perspective of CCH.

The pathological cellular mechanisms of CCH include mitochondrial dysfunction, impaired glucose metabolism, and autophagy dysfunction. Metabolic disturbance and neuroinflammation play pivotal roles in the initiation and progression of CCH, exacerbating neuronal death, myelin injury, as well as blood-brain barrier (BBB) disruption [7–10]. These complex processes must be controlled to help preserve neuronal survival and white matter integrity, as they underlie the prognosis of neurological function. Despite recent investigations into its related experimental and clinical neuroscience, few strategies have been identified to impede CCH-induced brain injury.

Irisin, a 112-amino acid polypeptide first discovered in skeletal muscle, is derived from the precursor fibronectin type III domain-containing protein 5 (FNDC5) [11, 12]. Contraction of skeletal muscles by physical exercise promotes FNDC5 gene expression, leading to the release of irisin into circulation following its cleavage from FNDC5 [12]. Irisin was first identified for its ability to induce the transformation of white adipocytes into brown adipocytes, facilitating rapid glucose consumption and fat burning [11]. Accumulating advanced investigations revealed abundant level of irisin in brain tissue, with varied degrees of expression in neurons and glial cells [13–16]. Under physiological conditions, irisin mediates the maturation-promoting effects of exercise on newborn neurons in the dentate gyrus, as well as the expression of brain-derived neurotrophic factor in hippocampal neurons [15, 17]. On the other hand, altered metabolism of irisin is implicated in the progression of neurodegenerative and cerebrovascular diseases. For instance, reduced level of irisin was observed in Alzheimer’s disease (AD) and acute ischemic stroke patients [18–22]. And serum irisin levels can serve as an important predictive factor for post-stroke neurological recovery, post-stroke depression, and prognosis [20–22]. Elevating irisin levels can ameliorate synaptic plasticity and memory impairment in AD mice, whereas blocking the peripheral or central expression of irisin can diminish the neuroprotective effects of exercise on AD mice [17]. Additionally, studies have found that exogenous administration of irisin can significantly improve the cerebral inflammatory response and reduce neurological dysfunction in mice with subarachnoid hemorrhage and cerebral hemorrhage [23–25].

However, current investigations regarding irisin metabolism into cerebrovascular diseases mainly focused on acute stroke, with limited investigation into CCH. By contrast to acute stroke with abrupt onset, CCH is characterized by a more insidious one, incurring negative impact on brain function and peripheral systems through a gradual process [26–28]. A decrease in serum irisin levels has been found in VaD patients and can serve as a biological marker for cognitive decline [29]. Muscle homeostatic function needs to be maintained by a positive brain-muscle communication [30]. It is likely that CCH could cause a metabolic change of irisin expression.

The role of physical exercise in the prevention and treatment of various neurological diseases, including AD, Parkinson’s disease, and stroke, has been widely recognized, and the metabolism of irisin may be one of the key regulators of the peripheral-central interplay [18, 21, 22, 31, 32]. Therefore, it is likely that irisin metabolism could be involved in the pathophysiological process of CCH. In the present study, a bilateral common carotid artery stenosis (BCAS) mouse model was adopted to assess peripheral and central irisin levels following relatively modest but prolonged brain injury caused by CCH. Our results provide further evidence that manipulating endogenous irisin expression through forced aerobic exercise can improve CCH-induced cognitive decline, anxiety- and depression-like behaviors through anti-inflammatory effects and attenuation of hippocampal apoptosis.

## Methods

### Animals

Male C57BL/6 mice (8 weeks old, weighing 25–30 g) were purchased from the First Affiliated Hospital of Sun Yat-sen University. Animals were housed in groups of 4–5 in a specific pathogen-free (SPF) facility at 21 ± 1 °C and 50 ± 5% humidity, under a 12-h light/dark cycle. Food and water were available *ad libitum*. Mice were randomly and blindly assigned to experimental or control groups. All experimental procedures were approved by the Animal Care and Use Committee of the First Affiliated Hospital of Sun Yat-sen University.

### Experimental design

**Fig. 1:**
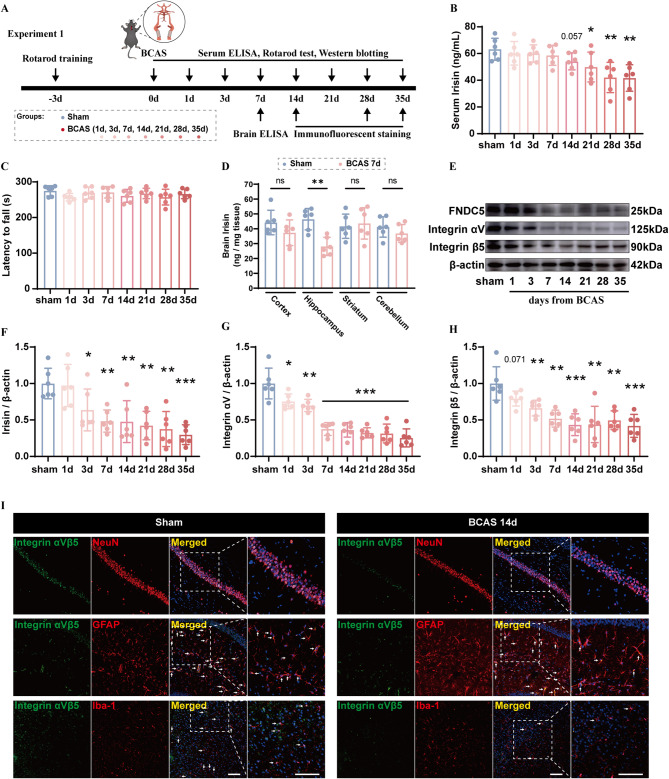
Expression profile of irisin and integrin αV and β5 after CCH. **A** Timeline of the experiment 1 (BCAS, bilateral common carotid arteries stenosis. ELISA, enzyme-Linked immunosorbent assay). **B** Quantification of serum irisin by ELISA (*n* = 6/group). **C** Latency to fall in Rotarod test (*n* = 6/group). **D** Quantification of irisin in various brain regions by ELISA (*n* = 6/group). **E-H** Western blot analysis of time-course protein levels of fibronectin type III domain-containing protein 5 (FNDC5), integrin αV, and β5 in the hippocampus after CCH, with quantification (band intensity normalized to β-actin) (*n* = 6/group). **I** Representative immunofluorescence images of integrin αVβ5 in hippocampal neurons, astrocytes, and microglia before and 14 days after CCH. Scale bar, 100 μm. Student’s t test (**B–H**). The data represent the mean ± SD, *p* < 0.05 was set as the threshold for significance. * *p* < 0.05, ** *p* < 0.01, *** *p* < 0.001 compared to the sham group

Mice were blindly and randomly assigned to experimental or control groups, as shown in Figs. 1A and 4A, and Additional File 1: Fig. S1. Summaries of the experimental groups, animal numbers, and mortality rates are provided in Additional File 1: Tables S1–S3.

### Experiment 1

Forty-eight mice were randomly divided into eight groups (sham, and 1, 3, 7, 14, 21, 28, and 35 days after BCAS; *n* = 6 per group). Three days before surgery, the mice underwent Rotarod training. After surgery, tail vein blood was collected from sham and BCAS mice at the corresponding time points for serum irisin quantification by ELISA. Subsequently, the mice underwent Rotarod testing to evaluate the direct impact of CCH on skeletal muscle and motor function. Then they were sacrificed for brain tissues collection, protein extraction, and western blotting analysis. Among them, brain irisin levels at varied regions were quantified by ELISA in sham and BCAS 7 d groups (*n* = 6 for each group). An additional 20 mice were randomly divided into four groups (sham, 14, 28, and 35 days post-BCAS; *n* = 5 per group) for immunofluorescence staining (Fig. 1A).

### Experiment 2.1

Seventy-two mice were randomly divided into six groups: sham + control (Ctrl), sham + forced aerobic exercise (FAE), BCAS + Ctrl, BCAS + FAE, BCAS + FAE + DMSO, and BCAS + FAE + Cilengitide trifluoroacetate (CT) (*n* = 12 per group). Five days before surgery, mice underwent either control or swimming training. From day 2 to 27 after surgery, mice received either standard care or a daily swimming protocol (1 h/day, 5 days/week), with careful postoperative monitoring to prevent infection. Meanwhile, the BCAS + FAE + DMSO and BCAS + FAE + CT groups received daily intraperitoneal injections of DMSO or CT (10 mg/kg). On day 28 post-surgery, tail vein blood was collected for serum irisin quantification by ELISA (*n* = 10 per group). Behavioral tests were conducted in the following order to avoid interference: Novel Object Recognition Test (NORT) on days 29–30, Y-maze Test (YMT) on day 31, Open Field Test (OFT) on day 32, Elevated Plus Maze Test (EPMT) on day 33, and Tail Suspension Test (TST) on day 35 (*n* = 10 per group). After behavioral assessment, mice were sacrificed for either (1) brain tissue collection and western blotting analysis with irisin quantification by ELISA (*n* = 6), or (2) coronal brain section preparation and immunofluorescence staining (*n* = 6) (Fig. 4A).

### Experiment 2.2

Seventy-two mice were randomly divided into six groups: sham + Ctrl, sham + FAE, BCAS + Ctrl, BCAS + FAE, BCAS + FAE + DMSO, and BCAS + FAE + CT (*n* = 12 per group). Five days prior to surgery, all mice underwent either control handling or swimming training. Following surgery, animals continued to receive either standard housing (control) or daily swimming sessions (1 h/day, 5 days/week) from day 2 to day 13 post-surgery. All mice received routine postoperative care to prevent infection and minimize stress. In the BCAS + FAE + DMSO and BCAS + FAE + CT groups, mice were administered daily intraperitoneal injections of either DMSO or CT (10 mg/kg) throughout the swimming period. On day 14 post-surgery, all mice were sacrificed for brain tissue collection. Samples were either processed for protein extraction and western blot analysis (*n* = 6 per group), or used to prepare coronal brain sections for immunofluorescence staining (*n* = 6 per group) ( Additional File 1: Fig. S1).

### Chronic cerebral hypoperfusion (CCH) mouse model

CCH surgery was conducted by bilateral common carotid artery stenosis (BCAS) as previously described [8]. Briefly, after habituation of at least 7 days, mice were anesthetized with 1–2% isoflurane (RWD, Shanghai, China) in a 30% O_2_ / 70% N_2_ mixture. Through a midline incision, the left and right common carotid artery (CCA) were exposed and separated carefully from the sheaths and vagus nerves. Then microcoils (microcoil specifications: piano wire diameter 0.08 mm, internal diameter 0.18 mm, coiling pitch 0.5 mm, and total length 2.5 mm; Sawane Spring Co Ltd, Japan) were twined gently around each CCA by rotation. The sham group underwent the same surgical procedures except implantation of microcoils. During the entire operation, rectal temperature of the mice was maintained at 36.5–37.5 °C using a temperature-regulated heating pad. After the skin incision was sutured closed, the mice recovered from anesthesia and were returned to their cages. Postoperative animals were kept in individually ventilated cages, monitored twice daily, and administered antibiotics (enrofloxacin, 10 mg/kg) and analgesics (meloxicam, 2 mg/kg) via subcutaneous injection for three days as per institutional animal care guidelines to minimize infection and discomfort.

### Forced aerobic exercise in mice

To increase endogenous level of irisin, aerobic exercise in mice was performed as previously described with some modification [18]. In brief, mice were placed in an open clear Plexiglas cylindrical barrel (60 cm tall, 30 cm wide) containing 40 cm of water at 30–32 °C. To minimize water-induced stress, mice underwent a short adaptation period prior to the formal training, during which they were gradually exposed to shallow water to reduce novelty-induced stress. And mice were adapted to swimming for 10–15 min each day for 2 days. The swimming duration was progressively increased, reaching 60 min on average by the fifth day of training. Forced aerobic exercise (FAE) was conducted for five consecutive days in the middle of the experimental week, during the light phase. A 5-day-per-week schedule provides an adequate training load while allowing 2 days of recovery per week, which helps reduce cumulative physiological stress and ensures reproducibility. After swimming, they were removed and dried before being returned to their home cages.

### Drug administration

Cilengitide trifluoroacetate (10 mg/kg, Selleck, USA), a selective inhibitor of integrin αV and β5, was dissolved in DMSO and intraperitoneally injected daily during forced aerobic exercise (Fig. 4A and Additional file 1: Fig. S1) [23].

### Enzyme-linked immunosorbent assay (ELISA)

Brain and plasma levels of irisin were quantified using mouse-irisin kits (Phoenix Pharmaceutical, Burlingame, CA) according to the manufacturer’s instructions.

### Behavioral tests

Before testing, mice were placed in the testing room, while still in their home cages, for 30 min of habituation to minimize the effects of novelty or stress. Behavioral tests were conducted between 9:00 AM and 17:00 PM. The facilities were cleaned with 75% ethanol between each trial. The test results were analyzed using the Xmaze video tracking system (Shanghai XinRuan Information Technology Co., Ltd., China) or scored manually.

### Rotarod test

To assess potential deficits in motor coordination and balance induced by chronic cerebral hypoperfusion, the rotarod test was conducted at various time points following BCAS surgery. Rotarod test was conducted using a 47,650 Mouse Rota-Rod facility (Ugo Basile Srl, Varese, Italy). Briefly, rods began to rotate at a speed of 5 r/min and gradually increased to 40 r/min within 300 s. With an interval of 15 min, 3 trials were arranged for each mouse to record its falling latency. Data were expressed as the mean value from the trials. Mice were pre-trained 3 days before surgery and tested 1, 3, 7, 14, 21, 28, 35 days post surgery (Fig. 1A).

### Open field test (OFT)

In order to assess spontaneous locomotor activity and exploratory behavior of mice, the open field test was performed in an arena (40 cm×40 cm×40 cm). Each mouse was placed in the center and allowed to explore the arena for 10 min. The total distance, average speed, the duration and distance in and out of the central area were recorded.

### Y maze test (YMT)

The Y-maze test was performed to evaluate spatial working memory based on the natural tendency of rodents to explore novel environments. The percentage of spontaneous alternation behavior is considered a reliable indicator of hippocampus-dependent cognitive function. The Y maze consisted of three white opaque plexiglass arms (30 cm×6 cm×15 cm) positioned at 120° extending from a central platform. Each mouse was placed in the center platform and allowed to freely explore for 10 min. An arm entry was defined as the point at which the mouse fully entered an arm with all four limbs. The alternation rate was calculated by software as follows: actual alternation / maximum alternation*100%. The actual alternation value was the number of times that the mouse went into the three arms in a row. The maximum alternation value was the total number of arm entries minus 2.

### Novel object recognition test (NORT)

The novel object recognition test was conducted to evaluate recognition memory based on the natural tendency of rodents to explore novel objects more than familiar ones. The discrimination index, derived from exploration time, is used as a sensitive indicator of cognitive performance, particularly in assessing hippocampal- and perirhinal cortex-dependent memory. An open chamber (40 cm×40 cm×40 cm) was used for this test. During the adaptation stage, each mouse was allowed to explore the empty chamber for 10 min. In the training session conducted 24 h later, each mouse was given 10 min to explore two identical objects (object A and A’) placed at two corners of the chamber (both 10 cm from the corner). Four hours later in retention phase, one of the objects was replaced with a novel and differently shaped one (object B). The mice were then allowed to explore freely for another 10 min. The percentage of time spent exploring the familiar object and the novel object were calculated. Exploration by mice was defined as any activity performed within 2 cm radius around the object.

### Elevated plus maze test (EPMT)

The elevated plus maze test was used to assess anxiety-related behavior based on the natural aversion of rodents to open and elevated spaces. Increased time spent in the open arms is generally interpreted as a reduction in anxiety, while preference for the closed arms reflects anxiety-like behavior. The behavioral apparatus consisted of two open arms and two closed arms (width 5 cm × length 30 cm). The labyrinth was 50 cm above ground. The mice were placed individually in the center of the maze facing an open arm and allowed to freely explore for 5 min. The time spent and distance traveled in each arm were recorded.

### Tail suspension test (TST)

The tail suspension test (TST) was conducted to evaluate depression-like behavior based on behavioral despair. In this test, increased immobility time is interpreted as a marker of depressive-like states, while decreased immobility suggests antidepressant-like effects or improved emotional status. The mice were suspended from a rod (50 cm tall, 30 cm wide) 10–15 cm above the ground for 6 min. They were attached via adhesive tape placed 2–3 cm from the tip of the tail. The immobility time was recorded during the last 4 min.

### Western blot analysis

Western blot analysis was performed to examine the protein expression levels of irisin and its receptors, apoptosis-related proteins, pro-inflammatory markers of microglia and astrocytes, inflammatory cytokines, and autophagy-related proteins. Under deep anesthesia, mice were transcardially perfused with cold PBS. After harvesting brain samples, cerebral cortex and hippocampal tissues were quickly separated using sharp forceps on ice under a microscope, then collected into a microtube and frozen at − 80 °C until protein extraction.

The mitochondrial proteins in the hippocampal region were extracted from the fresh brain tissue using a tissue mitochondria isolation kit (C3606, Beyotime, China) according to the manufacturer’s instructions.

To acquire total protein, cerebral cortex or hippocampal tissues were homogenized in lysis buffer (9803 S, Cell signaling Technology, Danvers, MA, USA) containing 100 mM phenylmethyl sulfonyl fluoride (1:100), a protease inhibitor cocktail (1: 20, Cat# P-2714; Sigma, USA), and a phosphatase inhibitor cocktail (1:10, 04906837001, Roche, Mannheim, Germany). Each sample was disrupted using an ultrasonic homogenizer and centrifuged at 15,000 rpm for 15 min at 4 °C. The supernatant was collected and assessed with a BCA protein assay kit (ShareBio, China) for protein quantification. Equal amounts of protein (35–50 µg) were separated by sodium dodecyl sulfate-polyacrylamide gels (SDS-PAGE) of appropriate concentrations and transferred to polyvinylidene difluoride (PVDF) membranes (Merck, Germany). After blocking with 5% skim milk (P0216, Beyotime, China) at room temperature for 1 h, PVDF membranes were incubated with primary antibodies overnight at 4 °C. The primary antibodies used in this study included rabbit monoclonal anti-FNDC5 (1:1000, ab174833; Abcam, England), rabbit monoclonal anti-integrin αV (1:1000, ab179475; Abcam, England), rabbit monoclonal anti-integrin β5 (1:1000, #3629; Cell Signaling Technology, USA), rabbit polyclonal anti-caspase 3 (1:1,000, 19677-1-AP; Proteintech, China), rabbit monoclonal anti-Fc RII/III receptor (CD16) (1:1,000, ab211151; abcam, England), rabbit monoclonal anti-CD21 (C3d) (1:1,000, SC0681; HUABIO, China), rabbit polyclonal anti-Bcl 2 (1:1,000, 12789-1-AP; Proteintech, China), rabbit polyclonal anti-Bax (1:1,000, 50599-2-Ig; Proteintech, China), rabbit polyclonal anti-Bcl-XL (1:1,000, 26967-1-AP; Proteintech, China), rabbit monoclonal anti-cytochrome c oxidase subunit IV isoform (COXIV) (1:5,000, 66110-1-Ig; Proteintech, China), rabbit polyclonal anti-nitric oxide synthase 2, inducible (iNOS) (1:500, BA0362; Boster, China), rabbit polyclonal anti-CD86 (1:500, 13395-1-AP; Proteintech, China), rabbit polyclonal anti-TNF-alpha (1:500, 17590-1-AP; Proteintech, China), rabbit polyclonal anti-Sequestosome-1 (P62) (1:1000, 18420-1-AP; Proteintech, China), rabbit polyclonal anti-Beclin 1/BECN1 (1:500, PB9076; Boster, China), rabbit polyclonal anti-MAP1LC3B (1:1000, L7543; Sigma-Aldrich, USA), rabbit monoclonal anti-phospho-mTOR (pmTOR) (phospho S2448) (1:1,000, ab109268; abcam, England), rabbit polyclonal anti-mTOR (1:1000, 28273-1-AP; Proteintech, China), rabbit monoclonal anti-phospho-AMP-activated protein kinase α (pAMPKα) (1:1000, #2535; Cell Signaling Technology, USA), rabbit monoclonal anti-AMPKα (1:1000, #5831; Cell Signaling Technology, USA), mouse monoclonal anti-β-actin (1:5000, 66009-1-Ig; Proteintech, China). Then, after washing at least three times, PVDF membranes were incubated with peroxidase-conjugated goat anti-rabbit or goat anti-mouse secondary antibodies (1:5000, SA00001-2 and SA00001-1; Proteintech, USA) and visualized by a ChemiDoc MP System (Bio-Rad, USA). The quantification of protein expression was analyzed by ImageJ software (National Institutes of health, Bethesda, MD, USA) and the expression of target protein was normalized to β-actin, GAPDH, or COXIV levels.

### Immunofluorescence staining

Immunofluorescence staining was conducted to assess the colocalization of integrin αV and β5 with hippocampal neurons, microglia, and astrocytes, as well as to evaluate hippocampal neuronal loss, white matter injury, and the activation of microglia and astrocytes. In addition, TUNEL staining was used to detect neuronal apoptosis in the hippocampus. After fasting for 24 h, mice were perfused transcardially with ice-cold saline and 4% paraformaldehyde under deep anesthesia. The brains were harvested and post-fixed in 4% paraformaldehyde, and dehydrated with 20% sucrose and 30% sucrose in sequence. Then 25-µm coronal brain sections were prepared using a freezing microtome (HM525NX, ThermoFisher, USA). The brain sections were then washed with PBS, and blocked with 10% goat serum or donkey serum for 1 h, followed by overnight incubation at 4 °C with the following primary antibodies: mouse monoclonal anti-NeuN (1:500, 66836-1-Ig; Proteintech, USA), rabbit polyclonal anti-NeuN (1:500, 26975-1-AP; Proteintech, China), rabbit polyclonal anti-myelin basic protein (MBP) (1:500, 10458-1-AP; Proteintech, China), goat monoclonal anti-Iba-1 (1:500, ab289874; abcam, England), chicken polyclonal anti-GFAP (1:1000, ab4674; abcam, England), rabbit monoclonal anti-CD16 (1:50, ab211151; abcam, England), rabbit monoclonal anti-C3d (1:50, SC0681; HUABIO, China), rabbit monoclonal anti-integrin αV (1:200, ab179475; Abcam, England), rabbit monoclonal anti-integrin β5 (1:200, #3629; Cell Signaling Technology, USA). Then the sections were treated with the appropriate Alexa-Fluor-conjugated antibodies (1:1000, ab150073, ab150076, ab150132, ab150172; abcam, England) for 1 h at room temperature and coverslipped with DAPI-Fluoromount-G ( P0126, Beyotime, China). TUNEL staining of brain sections was conducted using a TUNEL apoptosis assay kit (C1088, Beyotime, China) according to the manufacturer’s instructions. Sections were observed and imaged under a microscope (Olympus). Images were analyzed using the ImageJ (version 1.8.0, NIH) software.

### Statistical analysis

The data were analyzed using GraphPad Prism 8 (version 8.0, La Jolla, CA, USA) and are presented as the mean ± standard deviation (SD). Normality and homogeneity of variances were confirmed prior to each parametric test. Comparisons of continuous variables were performed using two-tailed Student’s t-tests or one-way analysis of variance (ANOVA), followed by Turkey’s multiple comparisons test. In addition, relationships between serum irisin levels and significant behavioral parameters were evaluated using Pearson correlation analysis. A *p*-value < 0.05 was considered statistically significant. Effect sizes for correlation analyses are reported as *R²* values.

## Results

### CCH decreases hippocampal and peripheral level of irisin

A total of 70 mice were used in experiment 1 (Fig. 1A), among which 59 were subjected to BCAS. Two BCAS mice died and were excluded. The mortality rate of BCAS mice in Experiment 1 was 2.86%. None of sham mice died (Additional file 1: Table S1). Tail vein blood was collected from sham mice and BCAS mice, followed by serum irisin quantification using ELISA. And the results showed a stably elevated level of irisin after surgery until 14 days post BCAS (Fig. 1B). The low serum level of irisin persisted from day 21 to day 35 after BCAS (Fig. 1B), indicating a disrupted metabolism of irisin. Since peripheral irisin mainly derived from contracting skeletal muscles, Rotarod test was conducted after tail vein blood collection. Sham and BCAS groups had no significant difference regarding their latency to fall, thereby excluding the direct impact of CCH on skeletal muscle and motor system (Fig. 1C). In order to explore the effect of CCH on central irisin expression, we collected varied brain zones of sham mice and BCAS mice at a relatively early stage (7 days after surgery). Compared to sham mice, level of irisin at hippocampus of BCAS mice was significantly lower (*t* (10) = 4.739, *P* = 0.001) (Fig. 1D). Then we collected hippocampal tissues of sham mice and BCAS mice (from day 1 to day 35 after surgery) for protein extraction. Western blotting analysis confirmed a gradual decrease in hippocampal FNDC5 levels, the precursor of irisin, from day 3 to day 35 following BCAS (Fig. 1E, F). Integrin αV and β5 are receptors of irisin, and have been reported to express on neurons, microglia, and astrocytes [23, 33–35]. Both integrin αV and β5 exhibited obviously reduced expression after BCAS, even earlier than that of FNDC5 (Fig. 1E, G, H). In addition, colocalization of integrin αV and β5 and hippocampal neurons, microglia, and astrocytes was revealed by immunofluorescence staining, suggesting a possible effect of irisin on biological activities of hippocampal neurons, microglia, and astrocytes under CCH (Fig. 1I).

### CCH causes hippocampal neuronal apoptosis, white matter injury, and neuroinflammation in a time-dependent manner

**Fig. 2:**
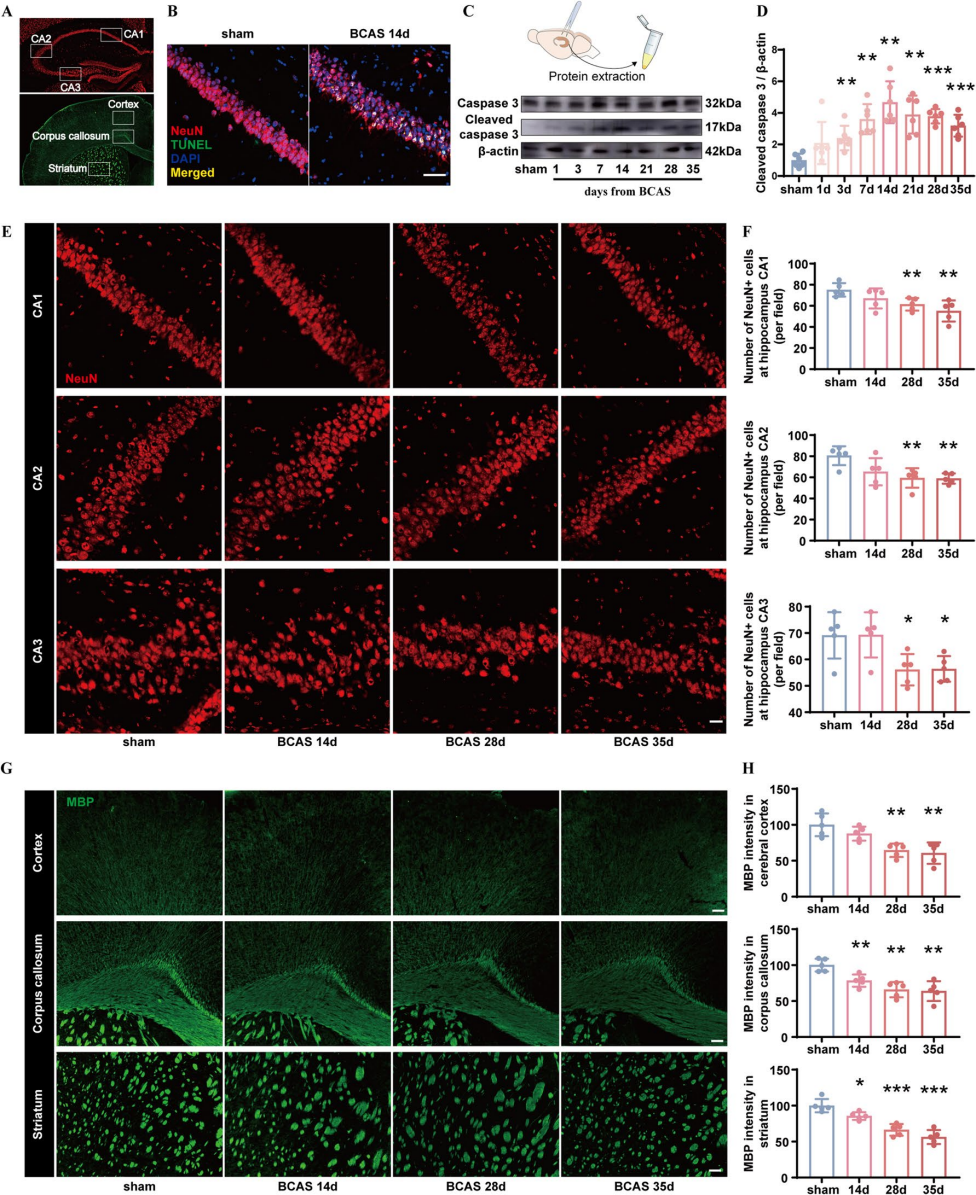
Hippocampal neuron apoptosis and white matter injury induced by CCH. **A** A schematic diagram for Cornu ammonis (CA) 1, CA2, CA3, cortex, corpus callosum, and striatum zones examined in the experiment. **B** TUNEL staining of hippocampal neurons. Scale bar, 50 μm. **C**,** D** Western blot analysis for apoptosis executor protein levels, including caspase 3 and cleaved caspase-3 expression in the hippocampus after CCH, with quantification (band intensity normalized to β-actin) (*n* = 6/group). **E**,** F** Representative immunofluorescence images of NeuN + hippocampal neurons in CA1, CA2, and CA3 zones, and their quantification per field (*n* = 5/group). Scale bar, 25 μm. **G**,** H** Representative immunofluorescence images of MBP + cortex, corpus callosum, and striatum zones, and their quantification of mean fluorescence intensity (*n* = 5/group). Scale bar, 100 μm. Student’s t test (**D**,** F**,** H**). The data represent the mean ± SD, *p* < 0.05 was set as the threshold for significance. * *p* < 0.05, ** *p* < 0.01, *** *p* < 0.001 compared to the sham group

Coronal brain sections of sham and BCAS mice were prepared for immunofluorescent staining, and BCAS mice displayed a higher number of TUNEL-positive apoptotic hippocampal neurons than sham mice (Fig. 2A, B). Hippocampal tissues from sham mice and BCAS mice (from day 1 to day 35 after surgery) were collected, followed by protein extraction and western blot analysis for cleaved caspase 3 (CC3), the crucial executor for cellular apoptosis. The expression of CC3 increased at day 3, peaked at day 14, and remained at a stably high level even 35 days after BCAS (Fig. 2B, C). In addition, brains of sham and BCAS mice (14, 28, and 35 days after surgery) were collected for immunofluorescent staining of NeuN-positive hippocampal neurons and myelin basic protein (MBP)-positive white matter (Fig. 2A). Compared to sham mice, numbers of NeuN + neurons in cornu ammonis (CA) 1, CA2 and CA3 regions were significantly decreased at day 28 and 35 in BCAS mice (Fig. 2D, E). And BCAS mice exhibited lower mean fluorescent intensity of MBP at cerebral cortex at day 28 and 35 post BCAS, and at corpus callosum and striatum as early as day 14 (Fig. 2F, G).

**Fig. 3:**
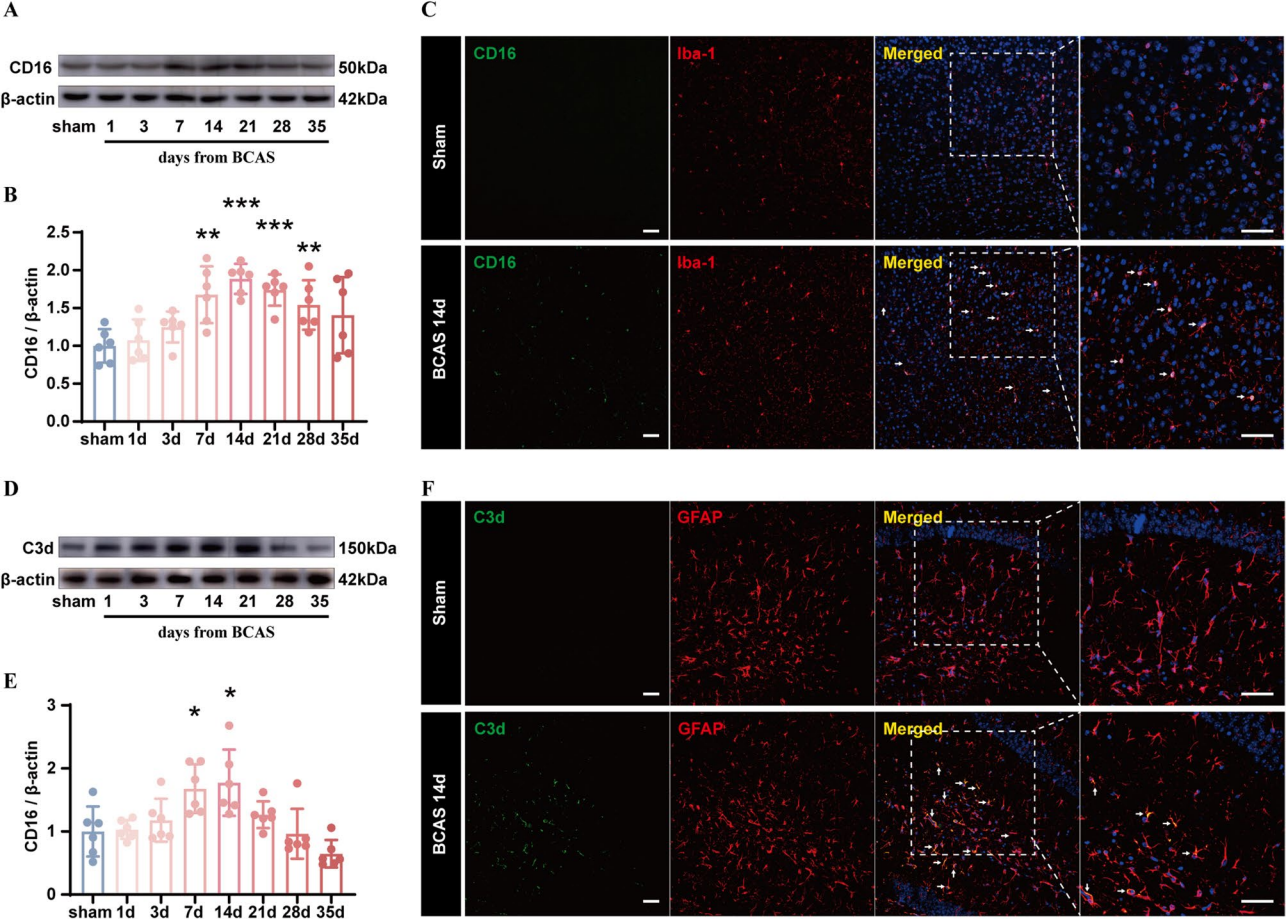
Neuroinflammation caused by CCH. **A**,** B** Western blot analysis of time-course protein levels of CD16 (pro-inflammatory markers for microglia) in the hippocampus after CCH, with quantification (band intensity normalized to β-actin) (*n* = 6/group). **C** Representative immunofluorescence images of pro-inflammatory microglia (Iba-1 + CD16+) in the hippocampus before and 14 days after CCH. Scale bar, 50 μm. **D**,** E** Western blot analysis of time-course protein levels of C3d (pro-inflammatory markers for astrocytes) in the hippocampus after CCH, with quantification (band intensity normalized to β-actin) (*n* = 6/group). **F** Representative immunofluorescence images of pro-inflammatory astrocytes (GFAP + C3d+) in the hippocampus before and 14 days after CCH. Scale bar, 50 μm. Student’s t test (**B**,** E**). The data represent the mean ± SD, *p* < 0.05 was set as the threshold for significance. * *p* < 0.05, ** *p* < 0.01, *** *p* < 0.001 compared to the sham group

Microglia and astrocytes proliferate, phagocytose debris, and play vital roles in the process of neuroinflammation caused by CCH [10, 36, 37]. Their dynamic activation was revealed as the expression of pro-inflammatory markers for microglia (CD16) and astrocytes (C3d) increased at days 3 and 7, respectively, and both peaked at day 14 (Fig. 3A–F). Then their expression started to decrease, with the down-regulation of C3d more rapidly (Fig. 3A–B, D–E).

### Forced aerobic exercise increases endogenous level of irisin

**Fig. 4:**
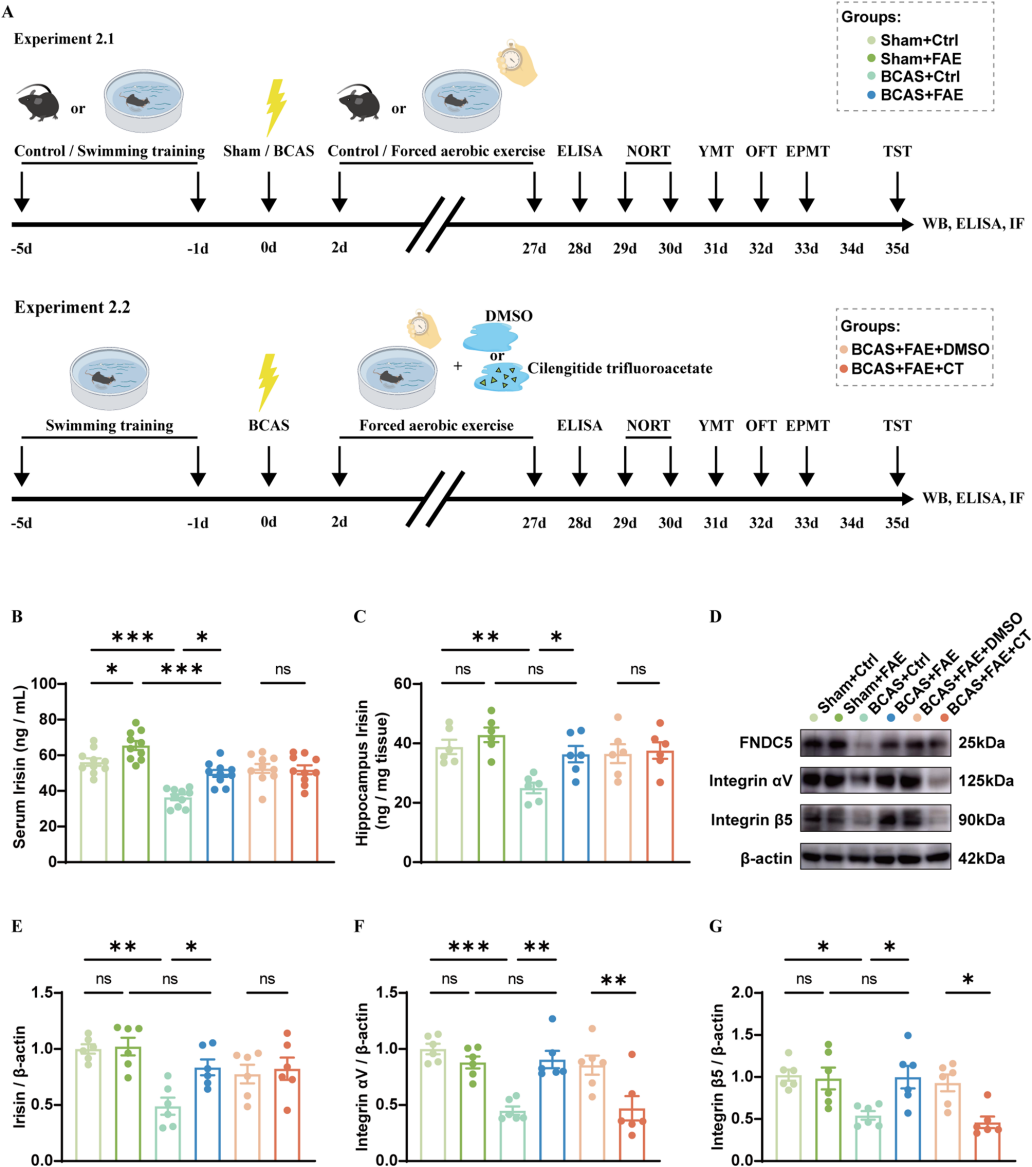
Expression profile of irisin and integrin αV and β5 after FAE. **A** Timeline of the experiment 2.1 (BCAS, bilateral common carotid arteries stenosis. ELISA, enzyme-Linked immunosorbent assay. WB, western blotting. IF, Immunofluorescence staining. NORT, new object recognition test. YMT, Y maze test. OFT, open-field test. EPMT, elevated plus maze test. TST, tail suspension test.). **B** Quantification of serum irisin by ELISA (*n* = 10/group). **C** Quantification of hippocampal irisin by ELISA (*n* = 6/group). **D-G** Western blot analysis for protein levels of fibronectin type III domain-containing protein 5 (FNDC5), integrin αV and β5 in the hippocampus, with quantification (band intensity normalized to β-actin) (*n* = 6/group). One-way ANOVA, Tukey post hoc test. The data represent the mean ± SD, *p* < 0.05 was set as the threshold for significance. * *p* < 0.05, ** *p* < 0.01, *** *p* < 0.001, ns, no significance, as indicated

Exercise has also been reported to ameliorate outcomes in neurological diseases like stroke, Alzheimer’s and Parkinson’s Disease, with accumulating evidence suggesting irisin as a critical regulator [38–41]. To investigate the effects of irisin on CCH-induced brain injury, mice were subjected to forced aerobic exercise (FAE) in order to increase their endogenous irisin. A protocol of daily swimming (1 h/day, 5 d/week) was adopted from day 2 to 27 after surgery, after which mice underwent a battery of behavioral tests arranged in a reasonable sequence to avoid interference (Fig. 4A). Moreover, cilengitide trifluoroacetate (CT), a selective inhibitor of integrin αV and β5, was administered to BCAS mice daily during FAE to further verify the mediator role of irisin, as depicted in Fig. 4A. The mortality rate of BCAS mice in Experiment 2.1 was 4.00% (Additional file 1: Table S2). The next day after FAE, tail vein blood was collected from mice, followed by serum irisin determination (Fig. 4A). The results of ELISA showed that FAE increased peripheral irisin levels of both sham and BCAS mice, either with CT administration or not (Fig. 4B). After behavioral tests, mice were sacrificed at day 35 to collect brain hippocampal tissues for histological experiments (Fig. 4A). ELISA and western blot analysis confirmed that FAE enhanced the expression of irisin and FNDC5 in hippocampus of BCAS mice as well (Fig. 4C–E). In addition, expression of integrin αV and β5 was significantly promoted in BCAS mice with FAE (Fig. 4D, F, G). However, this upregulation was effectively inhibited by CT treatment (Fig. 4D, F, G). The above findings suggested that FAE increased endogenous level of peripheral and central irisin.

### Elevated endogenous irisin alleviates cognitive decline and depressive-like behaviors after CCH

**Fig. 5:**
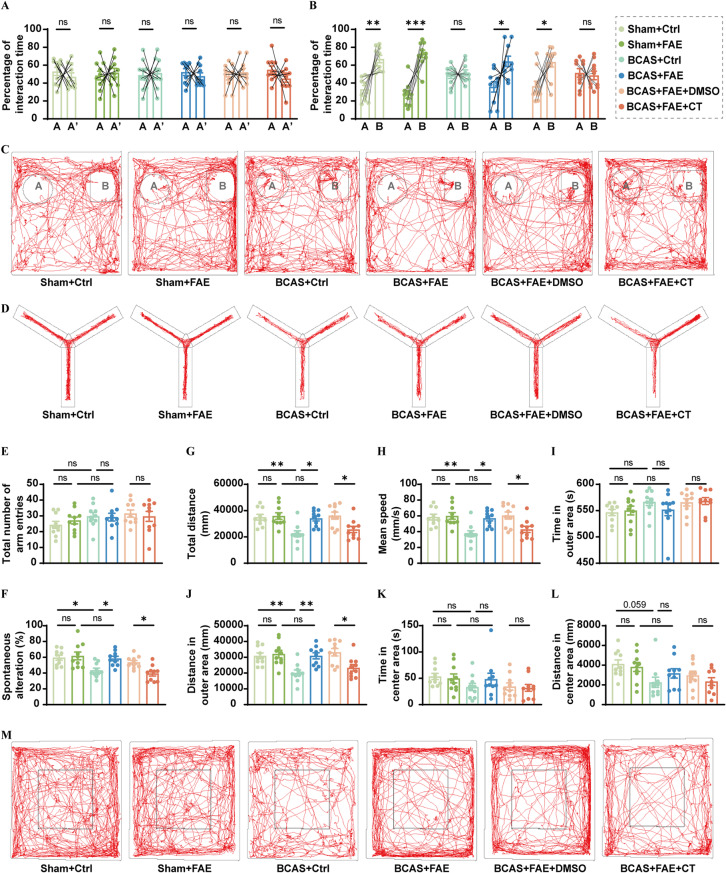
Effects of increasing endogenous irisin by FAE on cognitive outcomes in CCH. **A-C** Percentage of interaction time with two identical objects (object A and A’) in the training session (**A**), two different objects (object A and B) in the retention phase (**B**) of new object recognition test, and representative traces in the retention phase (**C**). **D-F** Representative traces in Y maze test (**D**), the total number of arm entries (**E**), and the spontaneous alteration rate (**F**). **G-M** Total travelled distance (**G**), mean speed (**H**), time in the outer area (**I**), travelled distance in the outer area (**J**), time in the center area (**K**), travelled distance in the center area (**L**), and representative traces (**M**) in open-field test (*n* = 10/group). Student’s t test (**A–B**) and One-way ANOVA, Tukey post hoc test (**E–L**). The data represent the mean ± SD, *p* < 0.05 was set as the threshold for significance. * *p* < 0.05, ** *p* < 0.01, *** *p* < 0.001, ns, no significance, as indicated

The behavioral tests in this study were performed for assessment of memory ability, spontaneous activity, anxiety- and depression-like emotion. In the novel object recognition test (NORT) conducted to evaluate recognition memory on days 29 and 30 post-surgery, all groups of mice showed no variation in their exploration tendencies toward two identical objects (object A and A’) during the training session (Fig. 5A). After replacing object A’ with a new and differently shaped one (object B) during the retention phase, the sham groups spent more time interacting with object B due to their novelty preference, while the BCAS control group did not show such a preference (*t*(18) = − 3.442, *P* = 0.003), indicating a recognition memory deficit (Fig. 5B, C). By contrast, FAE led to an increased interaction time with the novel object in BCAS mice (*t*(18) = − 3.424, *P* = 0.003), and this effect could be inhibited by CT treatment (*t*(18) = 0.412, *P* = 0.685) (Fig. 5B, C).

Similar effects were further confirmed in the Y-maze test (YMT) performed on day 31 post-surgery. Specifically, decreased spontaneous alternation was detected in BCAS control mice compared to sham control mice (*F* (5, 54) = 7.693, *P* = 0.015) (Fig. 5D–F). This sign of impaired short-term memory was ameliorated by FAE treatment (*F* (5, 54) = 7.693, *P* = 0.028), but reappeared upon co-administration with CT (*F* (5, 54) = 7.693, *P* = 0.042) (Fig. 5D–F). In the open-field test (OFT) conducted on day 32 post-surgery, under the same activity duration as sham control mice, BCAS control mice exhibited reduced total traveled distance and less distance traveled in the outer area, which were both improved by FAE treatment (Fig. 5G–M). However, this hypokinetic behavior re-emerged following CT administration in FAE-treated BCAS mice (Fig. 5G–M).

**Fig. 6:**
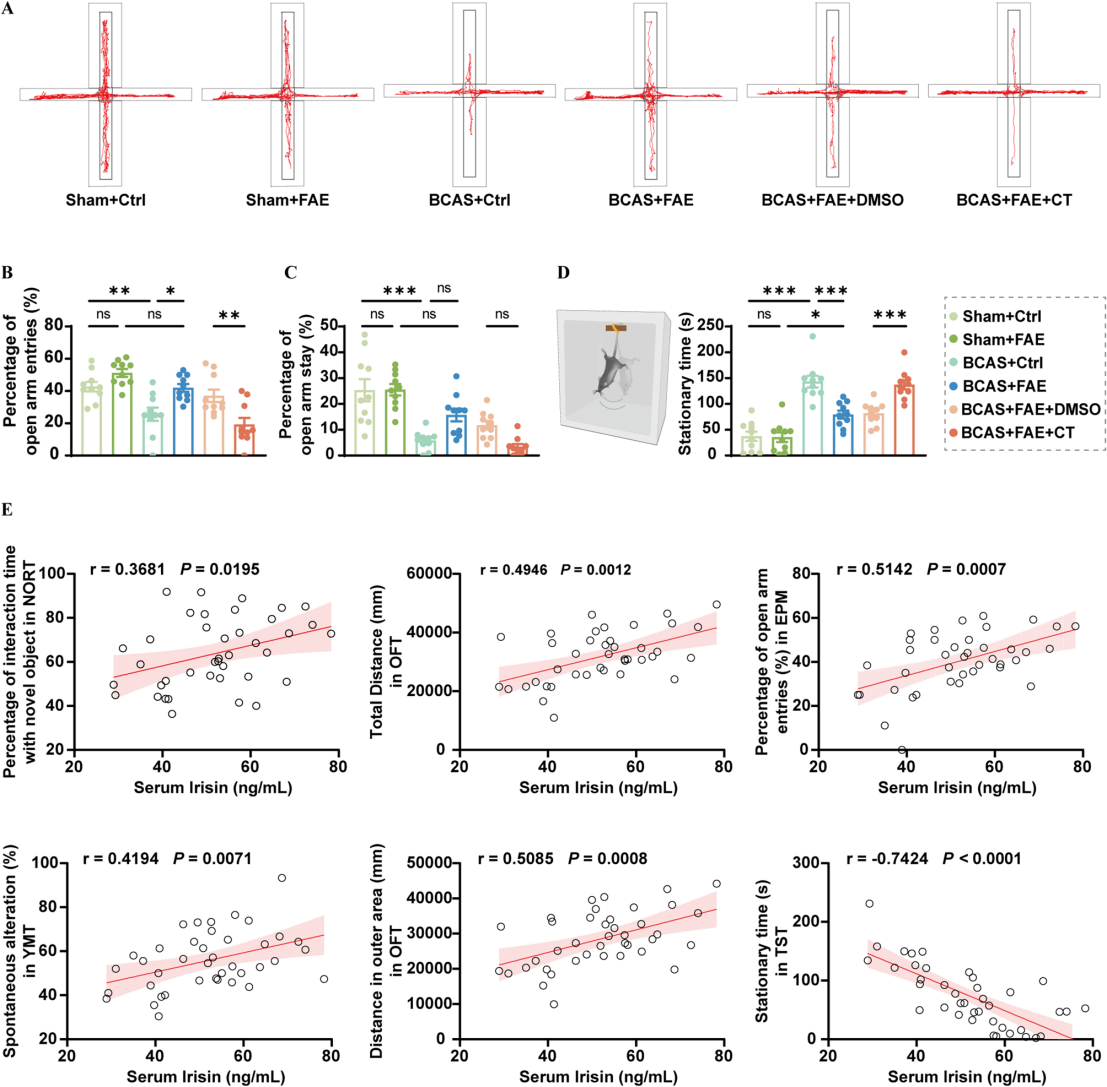
Effects of increasing endogenous irisin by FAE on anxiety- and depression-like behaviors caused by CCH, and correlation analysis between serum irisin levels and behavioral parameters. **A-C** Representative traces in elevated plus maze test **(A)**, the percentage of open arm entries **(B)**, and the percentage of open arm stay (**C**) (*n* = 10/group). **D** Duration of immobility in tail suspension test (**D**) (*n* = 10/group). **E** Correlation analysis between serum irisin levels and behavioral parameters with statistically significant differences. One-way ANOVA, Tukey post hoc test (**B–D**) and Pearson correlation (**E**). The data represent the mean ± SD, *p* < 0.05 was set as the threshold for significance. * *p* < 0.05, ** *p* < 0.01, *** *p* < 0.001, ns, no significance, as indicated

In the elevated plus maze test (EPMT) conducted on day 33 post-surgery, mice typically explore the open arms due to their natural curiosity, while BCAS control mice tend to remain in the closed arms, indicating an anxious state (Fig. 6A–C). FAE treatment alleviated anxiety-like behavior in BCAS mice, as indicated by an increased number of open arm entries (*F* (5, 54) = 12.26, *P* = 0.014) (Fig. 6A, B). In the tail suspension test (TST), sham mice continuously climbed and struggled in response to the inescapable stress. In contrast, BCAS mice exhibited behavioral despair, as indicated by significantly increased immobility time (*F* (5, 54) = 26.91, *P* < 0.001) (Fig. 6D). With FAE treatment, immobility time of BCAS mice was significantly decreased (*F* (5, 54) = 26.91, *P* < 0.001) (Fig. 6D). However, the anxiolytic and antidepressant effects of FAE were suppressed by CT (*F* (5, 54) = 26.91, *P* < 0.001) (Fig. 6A–D).

A previous investigation suggested that a decrease in serum irisin levels could serve as a biological marker for cognitive decline in patients with VaD, even after adjusting for risk factors [29]. In the present study, a significant though moderate positive correlation was observed between serum irisin levels in mice and their percentage of interaction time with the novel object in the NORT (*r* = 0.368, *P* = 0.020), the spontaneous alternation rate in the YMT (*r* = 0.419, *P* = 0.007), and the total distance traveled in the OFT (*r* = 0.495, *P* = 0.001). Serum irisin levels also showed a relatively strong correlation with the distance traveled in the outer area in the OFT (*r* = 0.509, *P* < 0.001), and the percentage of open arm entries in the EPM (*r* = 0.514, *P* < 0.001). Moreover, serum irisin levels were strongly and negatively correlated with immobility time in the TST (*r* = − 0.742, *P* < 0.001) (Fig. 6E). These behavioral data highlight the beneficial effects of endogenous irisin on anxiety, depression, and cognitive recovery following BCAS.

### Elevated endogenous irisin mitigates hippocampal neuronal apoptosis and withe matter injury post CCH

**Fig. 7:**
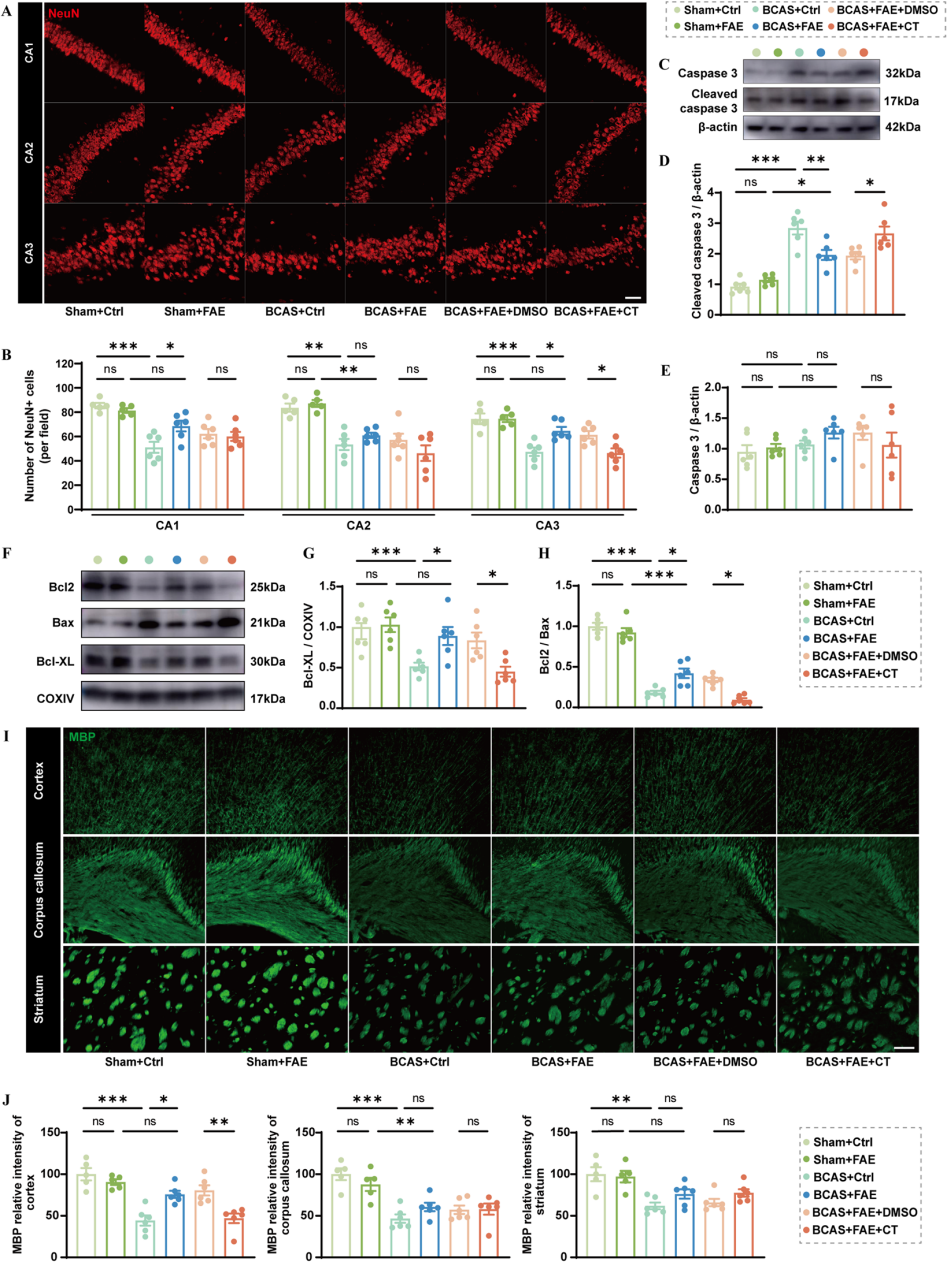
Effects of increasing endogenous irisin by FAE on hippocampal neuronal apoptosis and white matter injury triggered by CCH. **A**,** B** Representative immunofluorescence images of NeuN + hippocampal neurons in CA1, CA2, and CA3 zones, and their quantification per field (*n* = 6/group). Scale bar, 50 μm. **C-E** Western blot analysis of apoptosis executor protein levels, including caspase 3 and cleaved caspase-3 expression in the hippocampus after CCH, with quantification (band intensity normalized to β-actin) (*n* = 6/group). **F-H** Western blot analysis of apoptosis associated protein levels, including Bcl2, Bax, Bcl-XL expression in the hippocampus after CCH, with quantification (band intensity normalized to COXIV) (*n* = 6/group). **I**,** J** Representative immunofluorescence images of MBP + cortex, corpus callosum, and striatum zones, and their quantification of mean fluorescence intensity (*n* = 5/group). Scale bar, 100 μm. One-way ANOVA, Tukey post hoc test (**B–J**). The data represent the mean ± SD, *p* < 0.05 was set as the threshold for significance. * *p* < 0.05, ** *p* < 0.01, *** *p* < 0.001, ns, no significance, as indicated

Hippocampal degeneration and white matter lesions underlie cognitive decline in chronological aging and CNS diseases like AD, as well as depressive disorders [42–47]. Therefore, hippocampal neurons were examined using immunofluorescence staining. The results showed that the reduced number of NeuN-positive cells in the CA1 and CA3 regions was significantly restored following FAE treatment, indicating improved hippocampal neuron survival (Fig. 7A, B). Furthermore, the markedly increased expression of cleaved caspase-3 (CC3) in the hippocampus after BCAS was attenuated by FAE (*F* (5, 30) = 23.85, *P* = 0.006), as revealed by western blotting (Fig. 7C–E). In addition, mitochondrial proteins were extracted from the hippocampus for analysis of pro-apoptotic and anti-apoptotic markers. A decreased expression of the anti-apoptotic protein Bcl-XL (*F* (5, 30) = 7.990, *P* = 0.006) and a significantly reduced Bcl-2/Bax ratio (*F* (5, 30) = 87.30, *P* < 0.001) were observed in the hippocampus of BCAS control mice (Fig. 7F–H). FAE treatment increased the expression of Bcl-XL (*F* (5, 30) = 7.99, *P* = < 0.05) and the Bcl-2/Bax ratio (*F* (5, 30) = 87.30, *P* = 0.003), indicating inhibition of hippocampal apoptosis (Fig. 7F–H). However, hippocampal neuron survival was reduced, and neuronal apoptosis was exacerbated in FAE-treated BCAS mice that were co-administered with CT (Fig. 7A–H). On the other hand, FAE treatment improved the decreased mean fluorescence intensity of myelin basic protein (MBP) in the cortex after BCAS (*F* (5, 28) = 15.95, *P* = 0.005), but not in the corpus callosum (*F* (5, 28) = 10.88, *P* = 0.555) or striatum (*F* (5, 28) = 7.499, *P* = 0.454) (Fig. 7I, J). This protective effect was abolished upon co-treatment with CT (Fig. 7I, J).

### Elevated endogenous irisin attenuates microglia-mediated neuroinflammation

**Fig. 8:**
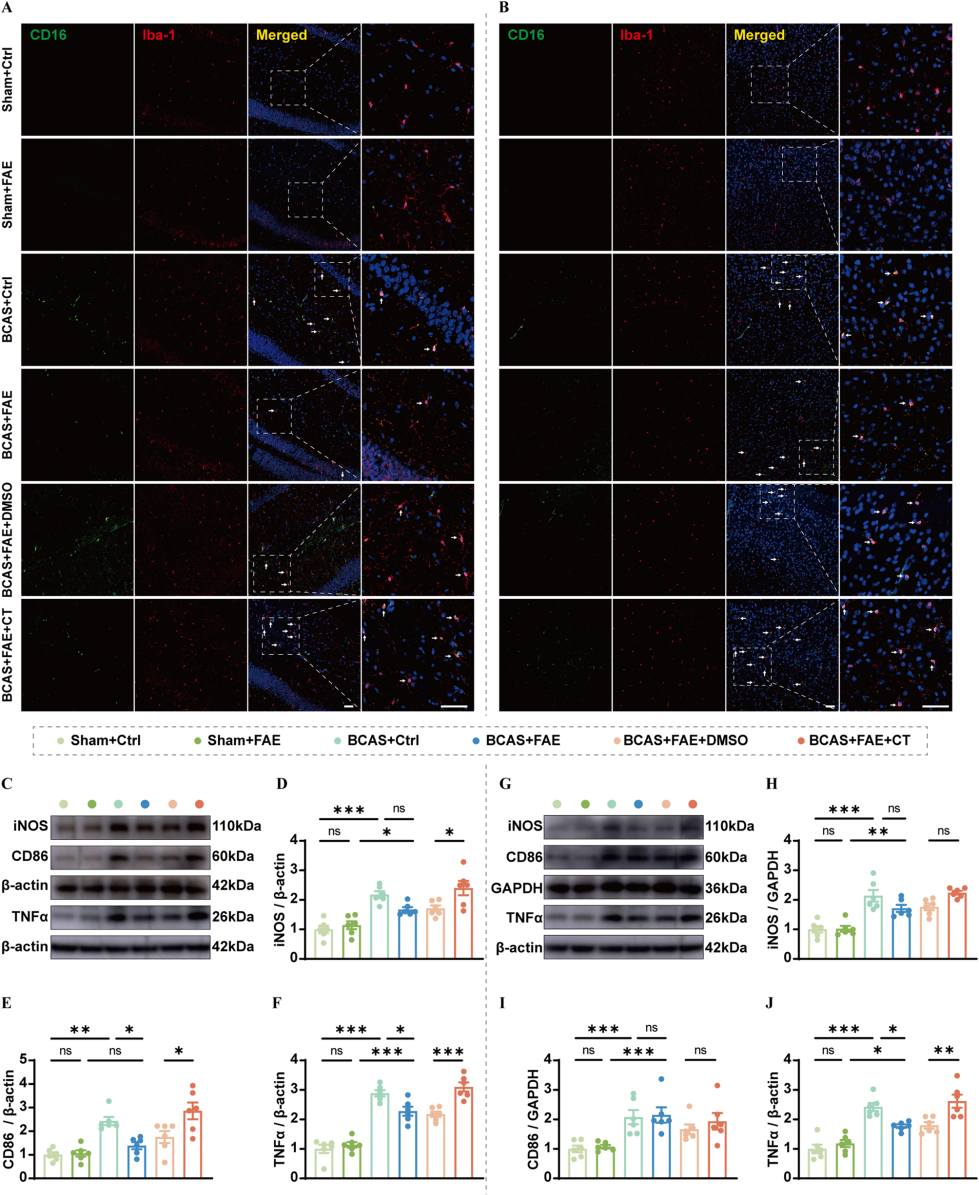
Effects of increasing endogenous irisin by FAE on microglia-mediated neuroinflammation induced by CCH. **A**,** B** Representative immunofluorescence images of pro-inflammatory microglia (Iba-1 + CD16+) in the hippocampus (**A**) and cortex (**B**). Scale bar, 50 μm. **C-J** Western blot analysis for pro-inflammatory protein levels, including iNOS, CD86, and TNFα in the hippocampus (**C-F**) and cortex (**G-J**), with quantification (band intensity normalized to β-actin) (*n* = 6/group). One-way ANOVA, Tukey post hoc test (**D–J**). The data represent the mean ± SD, *p* < 0.05 was set as the threshold for significance. * *p* < 0.05, ** *p* < 0.01, *** *p* < 0.001, ns, no significance, as indicated

Considering that the pro-inflammatory markers of microglia (CD16) and astrocytes (C3d) were both upregulated at a relatively early stage after BCAS (Fig. 3), Experiment 2.2 was conducted to investigate the effect of enhancing endogenous irisin levels via FAE on neuroinflammation (Additional file 1: Fig. S1). Briefly, sham and BCAS mice received either vehicle control or FAE treatment. Two additional groups of BCAS mice were treated with FAE in combination with either DMSO or CT, followed by sacrifice and tissue collection for histological analysis on day 14 post-surgery (Additional file 1: Fig. S1). A total of 36 mice were used in Experiment 2.2, with a mortality rate of 0.00% (Additional file 1: Table S3). Immunofluorescence staining revealed that FAE inhibited the BCAS-induced increase in pro-inflammatory microglia (CD16⁺Iba-1⁺) in the hippocampus, but not in the cerebral cortex (Fig. 8A, B). Neuroinflammation was further confirmed by Western blot analysis, which showed significantly upregulated expression of iNOS, CD86, and TNFα in both the hippocampus and cerebral cortex of BCAS control mice (Fig. 8C–J). Compared to the BCAS control group, FAE significantly reduced CD86 (*F* (5, 30) = 12.81, *P* = 0.017) and TNFα expression (*F* (5, 30) = 47.27, *P* = 0.022) in the hippocampus, as well as TNFα expression in the cerebral cortex (*F* (5, 30) = 20.85, *P* = 0.030), effects that were blocked by CT co-treatment (Fig. 8C–J). On the other hand, the number of activated astrocytes (C3d⁺GFAP⁺) and the protein level of C3d in the hippocampus of BCAS mice remained elevated despite FAE treatment (Additional file 1: Fig. S2). These findings suggest that FAE primarily attenuates microglia-mediated, rather than astrocyte-mediated, neuroinflammation in the hippocampus.

### Irisin promotes neuronal and microglial autophagy via integrin αVβ5/AMPK/mTOR signaling pathway

**Fig. 9:**
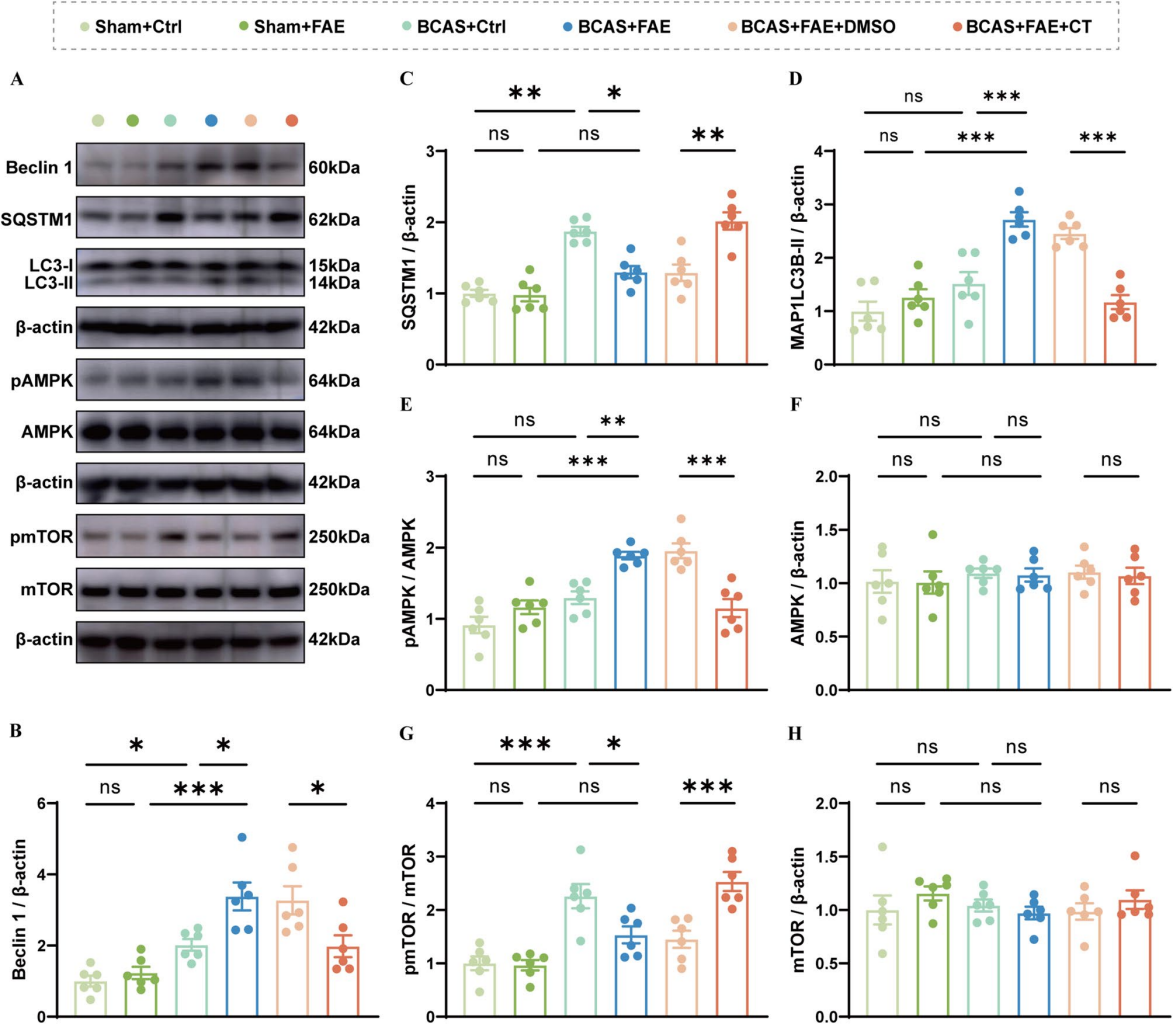
Effects of increasing endogenous irisin by FAE on autophagy and AMPK/mTOR pathway post CCH. **A-D** Western blot analysis for autophagy protein levels, including Beclin 1, SQSTM1, MAP1LC3B-I and MAP1LC3B-II in brain, with quantification (band intensity normalized to β-actin) (*n* = 6/group). **A**,** E-H** Western blot analysis for phosphorylation levels of AMPK and mTOR in brain, with quantification (band intensity normalized to β-actin) (*n* = 6/group). One-way ANOVA, Tukey post hoc test (**B–H**). The data represent the mean ± SD, *p* < 0.05 was set as the threshold for significance. * *p* < 0.05, ** *p* < 0.01, *** *p* < 0.001, ns, no significance, as indicated

Accumulating evidence has shown the potential of irisin in optimizing autophagy in peripheral organs [48–52]. However, its effect on autophagy in neurodegenerative diseases such as vascular dementia (VaD) remains unclear. Autophagy is a cellular self-digestion process essential for neural development and the maintenance of neuronal homeostasis [53]. Defective autophagy can lead to microglial activation [54, 55]. Therefore, the expression of autophagy-related proteins in the hippocampus of mice was examined. Compared to the sham group, BCAS resulted in increased expression of Beclin 1 (*F* (5, 30) = 12.35, *P* < 0.05), suggesting activation of autophagy initiation (Fig. 9A, B). Meanwhile, expression of SQSTM1 was elevated (*F* (5, 30) = 22.83, *P* < 0.001) and MAP1LC3B-II remained unchanged (*F* (5, 30) = 21.34, *P* = 0.207), indicating a potential blockage in the fusion of autophagosomes with lysosomes or in the degradation process (Fig. 9A, C, D). This suggests a bottleneck in the clearance phase of autophagy, leading to impaired autophagic degradation within cells. With FAE treatment, the expression of SQSTM1 decreased (*F* (5, 30) = 22.83, *P* = 0.002), while levels of Beclin 1 (*F* (5, 30) = 12.35, *P* = 0.022) and MAP1LC3B-II increased (*F* (5, 30) = 21.34, *P* < 0.001), as indicated by an increased number of MAP1LC3B-II-positive hippocampal neurons and microglia. This suggests a more active and efficient autophagic process, characterized by enhanced autophagosome formation and improved clearance of autophagic cargo (Fig. 9A–D; Additional file 1: Fig. S3). However, this effect of FAE was inhibited when co-treated with CT (Fig. 9A–D).

AMP-activated protein kinase (AMPK) signaling is a downstream target pathway after irisin binding with integrin αVβ5 receptor [23, 56]. AMPK orchestrates autophagy across various physiological and pathological contexts, such as nutrient deprivation, metabolic disorders, tumor cell suppression or survival, and neurodegenerative diseases [57–61]. Activated AMPK can suppress mTORC1 directly by phosphorylating Raptor, a key regulatory component of the mTORC1 complex, or phosphorylating Tuberous sclerosis complex 2 (TSC2), thereby enhancing the TSC1/2 complex’s inhibition of mTORC1 [62, 63]. FAE treatment elevated the phosphorylation level of AMPK (*F* (5, 30) = 18.13, *P* = 0.003), accompanied by a decrease in the phosphorylation level of mTOR (*F* (5, 30) = 15.66, *P* = 0.040) in BCAS mice (Fig. 9A, E-H). After blocking integrin αVβ5 receptor with CT, activation of AMPK was inhibited (*F* (5, 30) = 15.66, *P* < 0.001) and phosphorylation level of mTOR enhanced (*F* (5, 30) = 15.66, *P* < 0.001) (Fig. 9A, E-H). Therefore, the increased endogenous irisin level induced by FAE promoted neuronal and microglial autophagy via the integrin αVβ5/AMPK/mTOR pathway, facilitating the removal of damaged organelles and the accumulation of harmful substances, thereby potentially improving neuronal survival and attenuating microglial neuroinflammation.

## Discussion

**Fig. 10:**
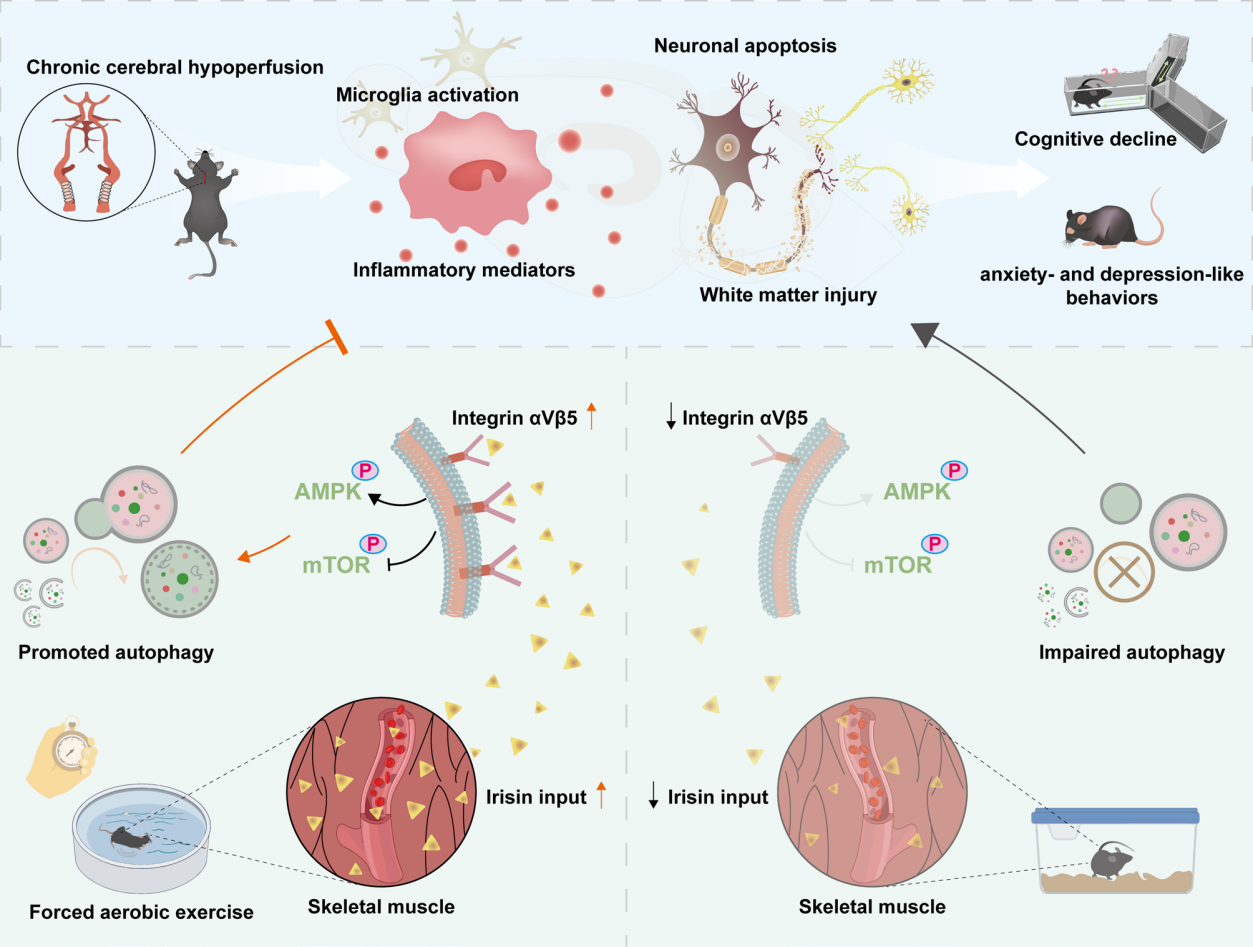
A schematic diagram for this study. Bidirectional communication exists between the brain and peripheral systems, involving metabolites such as irisin. Forced aerobic exercise can increase the production of irisin, which binds to integrin αVβ5, leading to enhanced AMPK phosphorylation and suppressed mTOR phosphorylation. This promotes neuronal and microglial autophagy, inhibits neuronal apoptosis and microglial activation, thereby improving neuronal survival and reducing white matter injury. Ultimately, this ameliorates cognitive decline as well as anxiety- and depression-like behaviors caused by CCH

The role of irisin in vascular dementia (VaD) remains unclear. In this study, from the pathophysiological perspective of VaD, we investigated irisin metabolism in a mouse model of chronic cerebral hypoperfusion (CCH). Although CCH did not affect the motor activity of mice, they exhibited reduced serum irisin levels from day 21 post-surgery. An earlier decrease in central irisin expression, especially in the hippocampus, was observed in CCH mice. Specifically, expression of the irisin precursor (FNDC5) in the hippocampus, as well as the irisin receptors (integrin αV and β5), were all suppressed within one week after CCH. The downregulation of integrin αVβ5 was localized in hippocampal neurons, microglia, and astrocytes. Irisin, mainly derived from contracting skeletal muscles, can cross the blood-brain barrier. After forced aerobic exercise, both serum concentration and hippocampal content of irisin were increased in CCH mice, accompanied by upregulation of integrin αV and β5 in the hippocampus. Through a battery of behavioral tests assessing cognition and emotion, we found that activation of integrin αVβ5 by increased endogenous irisin could attenuate recognition memory deficits, short-term memory decline, hypokinetic behavior, and anxiety- and depression-like behaviors caused by CCH. This effect was supported by the correlation between elevated serum irisin levels and behavioral improvements, and further confirmed by the relapse of behavioral deficits when co-treated with an integrin αVβ5 inhibitor. Moreover, aerobic exercise-induced irisin promoted autophagy in neurons and microglia via the integrin αVβ5/AMPK/mTOR signaling pathway, thereby alleviating hippocampal neuronal apoptosis and neuroinflammation following CCH. Taken together, our findings suggest that irisin serves as an important mediator in brain-muscle communication and has potential as a therapeutic target for secondary brain injury following CCH (Fig. 10).

Containing 112 amino acids, the sequence of irisin is completely conserved between human and rodent animals [11]. In the peripheral circulation, irisin is mainly derived from FNDC5, a glycosylated type I membrane protein, in contracting skeletal muscles. This cleaved and secreted form of FNDC5 participates in the regulation of various physiological activities, such as stimulating adipocyte browning and thermogenesis [11]. Acute cerebrovascular diseases, such as ischemic and hemorrhagic stroke, have been reported to reduce irisin expression [20, 23]. For example, in a mouse model of intracerebral hemorrhage, down-regulation of irisin in the brain and the circulation began almost simultaneously as early as 6 h after the onset [23]. The acute injury to neurons and white matter in the striatum caused by hemorrhage can lead to impaired motor activity and reduced skeletal muscle contraction, thereby decreasing the source of irisin. In our study, chronic cerebral hypoperfusion (CCH) did not affect the motor activity of mice, as evidenced by their performance in the Rotarod test. Consequently, serum irisin levels remained relatively high until day 14 post-surgery but started to decrease from day 21 onwards. A decrease in serum irisin levels has also been observed in patients with VaD [29]. Our previous studies revealed the interplay between CCH and peripheral organ like gut [28, 64]. It is speculated that the insidious impact of CCH on muscle may underlie the decreased serum irisin during the late stages of CCH, as positive brain-muscle communication is essential for maintaining normal muscle physiology, which deserves further investigation [30].

Studies have found that the mRNA expression of FNDC5 in the brain is the second highest among body organs [65–67]. Moreover, presence of irisin has been confirmed in multiple brain regions and cerebrospinal fluid (CSF) as well [19, 29, 68]. Reduced CSF irisin has been found to be positively correlated with the severity of AD pathology and dementia scores, especially in females, highlighting the use of irisin as a marker of the AD continuum [19]. Similarly, a previous study reported a significant correlation between decreased serum irisin levels and cognitive impairment in VaD patients [29]. In CCH mice, hippocampal irisin levels declined earlier than serum levels, and increased endogenous irisin levels were positively correlated with improvements in cognitive function, hypokinetic behavior, and anxiety- and depression-like behaviors, indicating the potential of central irisin as an early biomarker for predicting VaD.

Studies suggest that higher physical activity levels are associated with better brain structure and function, such as slower gray matter atrophy, improved white matter structural integrity, and enhanced brain network connectivity and synaptic integrity [69–73]. Ischemic stroke patients who previously did regular physical exercise would have a significantly reduced risk of disability at 5 years compared with those who did no exercise [74]. Patients with Parkinson’s disease of mild severity who underwent daily gamified aerobic exercise as part of their treatment regimen showed attenuated off-state motor signs [75]. On the other hand, physical exercise has been incorporated into multidomain interventions as promising non-pharmacological strategies to prevent all-cause dementia and AD [76–78]. One of the underlying molecular mechanisms driving this neuroprotection is likely to be the promoted expression of irisin. Endurance exercise could induce hippocampal *FNDC5* gene expression [15]. Blockade of either peripheral or brain FNDC5/irisin diminished the neuroprotective effect of physical exercise on synaptic plasticity and memory in AD mice [17, 18]. In our study, cognitive impairment of CCH mice with forced aerobic exercise treatment was significantly ameliorated, adding evidence supporting physical exercise for VaD protection. In addition, the mediator role of irisin in brain-muscle communication was validated, as inhibition of its receptor effectively reduced the beneficial effect of endurance exercise on VaD. Unlike acute stroke, it is likely that the relatively modest but prolonged nature of CCH could offer a time window for exercise training and persistence. And monitoring patients’ irisin levels can aid in developing personalized exercise training programs, thereby further optimizing therapeutic outcomes.

Stroke can be divided into acute ischemic stroke and hemorrhagic subtypes, the latter of which includes intracerebral hemorrhage (ICH) and subarachnoid hemorrhage (SAH). Accumulating evidence had demonstrated the neuroprotective effect of irisin in the secondary brain injury following stroke [23, 25, 79, 80]. Specifically, exogenous recombinant irisin, administered either intracerebroventricularly or intranasally, could reduce infarct size after acute ischemic stroke, decrease brain edema and improve neurological outcomes in ICH and SAH mice [23, 25, 79]. Moreover, neuronal apoptotic cell death and neuroinflammation were suppressed by irisin treatment [23, 25, 79]. However, few studies have explored the role of irisin in CCH. In our study, we found that irisin was downregulated post-CCH, and increasing its endogenous production facilitated its binding to the integrin αVβ5 receptor and the subsequent activation of the downstream AMPK pathway [34, 81, 82]. AMPK is a cellular energy sensor that is activated under low-energy conditions, characterized by decreased ATP levels and increased AMP/ADP ratios. It promotes energy production like glycolysis and fatty acid oxidation, while inhibiting energy-consuming processes, such as protein synthesis and lipogenesis. AMPK signaling has been found to participate in a variety of pathophysiological processes, such as mitigating cell apoptosis and strengthening autophagy [60, 82–84]. The activation of AMPK phosphorylates RPTOR/raptor, a key component of mTORC1, and TSC2 (TSC complex subunit 2), which acts as an upstream inhibitor of mTORC1 signaling. By phosphorylating RPTOR, AMPK promotes the induction of autophagy [63, 85–88]. In neurodegenerative diseases such as Alzheimer’s and Parkinson’s diseases, AMPK-induced autophagy contributes to the clearance of aberrant protein aggregates, enhancing neuronal viability [31, 89–91]. During sensorimotor recovery following traumatic brain injury, neural regeneration depends on optimized autophagy to accelerate the clearance of erroneously accumulated proteins in the axons [92]. In addition, by degrading inflammation-related proteins and senescent cells, microglial autophagy can reduce excessive neuroinflammation, preventing chronic inflammatory damage to the nervous system [54, 93]. Autophagy can limit the aberrant activation of microglia, reducing neuronal damage caused by neuroinflammation [94, 95]. In our investigation, BCAS resulted into the initiation of autophagy, but with a limited or hindered degradation function. This phenomenon may be associated with impairments in the later stages of autophagy, specifically lysosomal dysfunction. After FAE, a more active and efficient autophagic response was observed. Therefore, irisin could promote hippocampal neuronal survival, reduce apoptosis, and alleviate microglial activation following CCH by improving neuronal and microglial autophagy.

One limitation of this intervention is that the increased level of irisin for each mouse could not be normalized. It should be noted that research on irisin is still ongoing, and its pharmacokinetic properties in vivo, including its half-life, have not been fully elucidated. Future studies may clarify the metabolic process and key parameters of irisin. Irisin supplementation by FAE is consistent with the physiological process that the majority of irisin is cleaved and derived from the precursor FNDC5 in contracted skeletal muscles [11, 12]. Due to its small molecular weight, irisin can cross the BBB [96, 97]. Previous studies have shown that enhancing irisin levels by physical exercise is sufficient to improve both synaptic plasticity and memory defects in AD mouse models [17, 18]. VaD has a prolonged disease course, and aerobic exercise is a more reasonable approach to sustaining the continuous production and input of irisin. On the other hand, our data suggests that the increased endogenous irisin level triggered by FAE can promote neuronal and microglial autophagy. However, the specific stage of the autophagic flux affected by irisin, as well as the type of selective autophagy involved (mitophagy, reticulophagy/ER-phagy, or lysophagy), remains unclear. These will be further explored in future studies. Another limitation of this study is the exclusive use of male animals. However, research suggests that excluding female rodents solely due to estrous cycle variability may introduce bias and restrict the translational applicability of the findings [98, 99]. Additionally, stroke and several central nervous system diseases characterized by CCH predominantly affect the elderly. To address this, our future studies will involve aged mice or rats, with a particular focus on reproductively senescent females.

## Conclusion

The current study demonstrated that BCAS mice exhibited reduced peripheral and central levels of irisin. Enhancing endogenous irisin expression through forced aerobic exercise promoted neuronal and microglial autophagy via the integrin αVβ5/AMPK/mTOR pathway, thereby potentially improving neuronal survival and attenuating microglia-mediated neuroinflammation. Consequently, this intervention alleviated CCH-induced impairments in recognition memory, short-term memory, motor activity, and anxiety- and depression-like behaviors.

## Electronic supplementary material

Below is the link to the electronic supplementary material.

Supplementary Material 1

Supplementary Material 2

## Data Availability

No datasets were generated or analysed during the current study.

## Abbreviations

VaD: Vascular Dementia
CCH: Chronic Cerebral Hypoperfusion
BBB: Blood-Brain Barrier
FNDC5: Fibronectin type III domain-containing protein 5
AD: Alzheimer’s Disease
BCAS: Bilateral Common Carotid Artery Stenosis
FAE: Forced Aerobic Exercise
CD16: Fc RII/III receptor
COXIV: Cytochrome c Oxidase subunit IV isoform
iNOS: Nitric Oxide Synthase 2, inducible
pAMPKα: phospho-AMP-activated protein kinaseα
AMPKα: AMP-Activated Protein Kinaseα
MBP: Myelin Basic Protein
CC3: Cleaved Caspase 3
CA: Cornu Ammonis
CT: Cilengitide Trifluoroacetate
NORT: New Object Recognition Test
YMY: Y Maze test
EPMT: Elevated Plus Maze Test
TST: Tail Suspension Test
ICH: Intracerebral Hemorrhage
SAH: Subarachnoid Hemorrhage
