# Supplementary material for: Exercise-induced irisin ameliorates cognitive impairment following chronic cerebral hypoperfusion by suppressing neuroinflammation and hippocampal neuronal apoptosis
Source: J Neuroinflammation. 2025 Jun 28;22:168. doi: 10.1186/s12974-025-03493-5 (PMC12205520; doi:10.1186/s12974-025-03493-5)

Full unedited gel for Fig. 1E

FNDC5 expression after BCAS on 15% SDS-PAGE

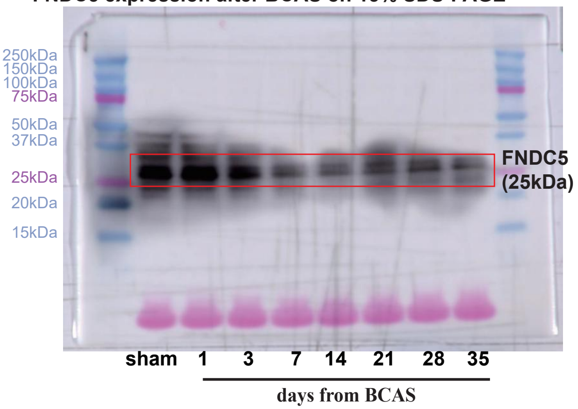


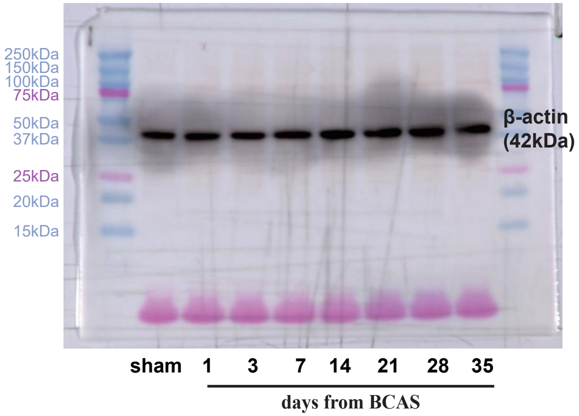


Integrin αV expression after BCAS on 4%-20% SDS-PAGE

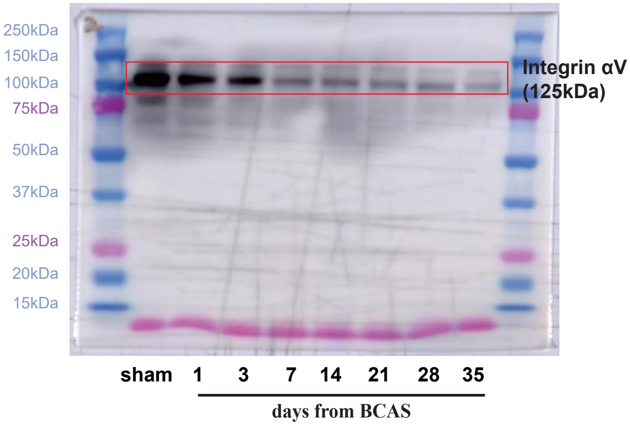


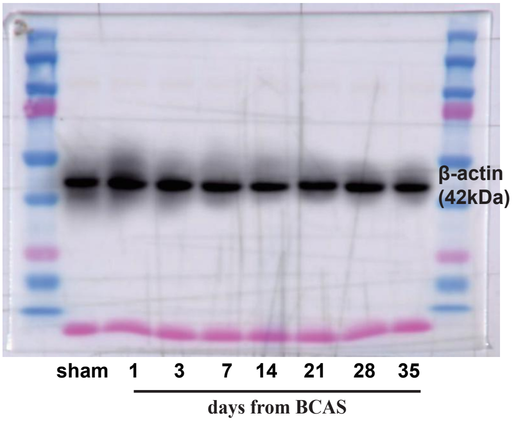


Integrin β5 expression after BCAS on 4%-20% SDS-PAGE

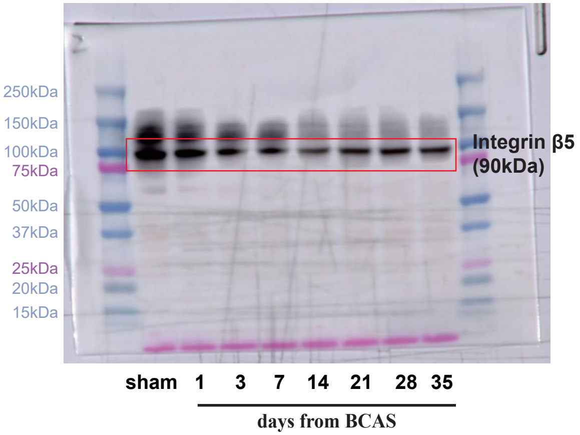


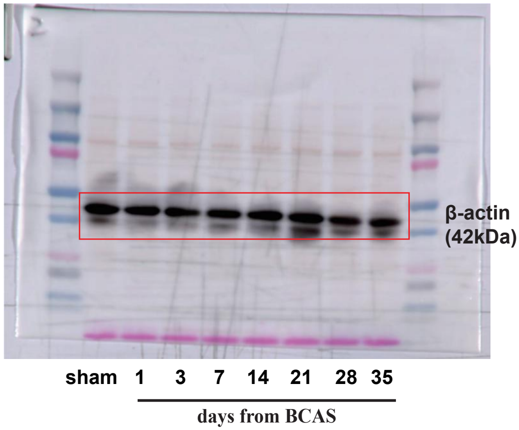

Full unedited gel for Fig. 2C

Caspase 3, Cleaved caspase 3 expression after BCAS on 15% SDS-PAGE

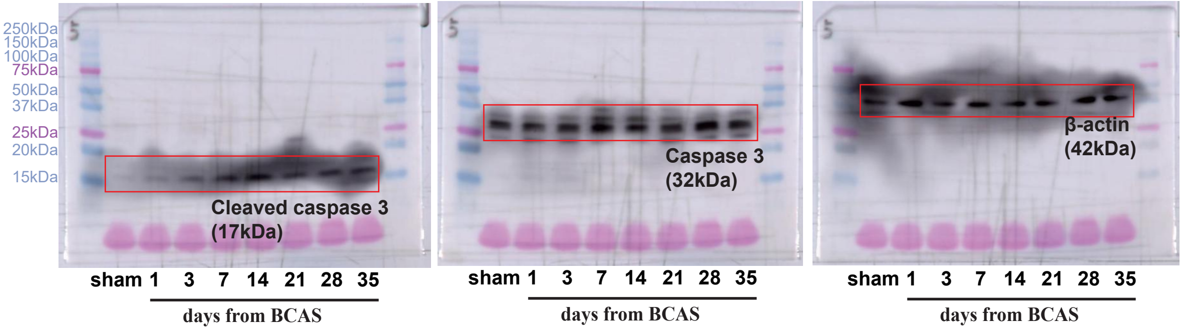

Full unedited gel for Fig. 3

CD16 expression after BCAS on 4%-20% SDS-PAGE

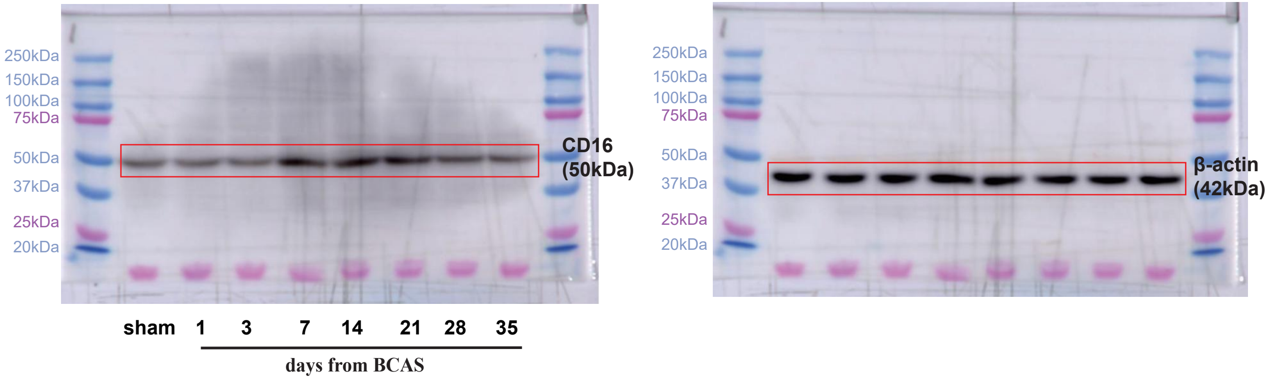


C3d expression after BCAS on 4%-20% SDS-PAGE

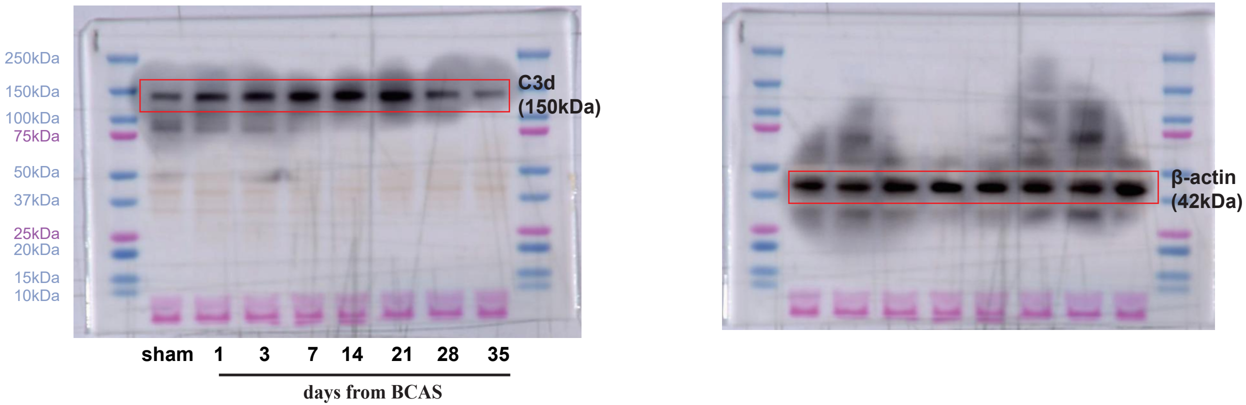

**Full unedited gel for Fig. 4D**

**FNDC5 expression on 15% SDS-PAGE**

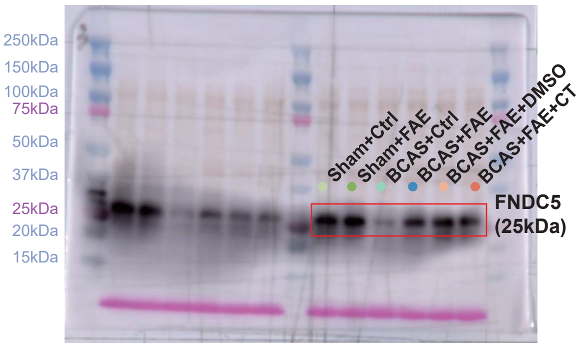


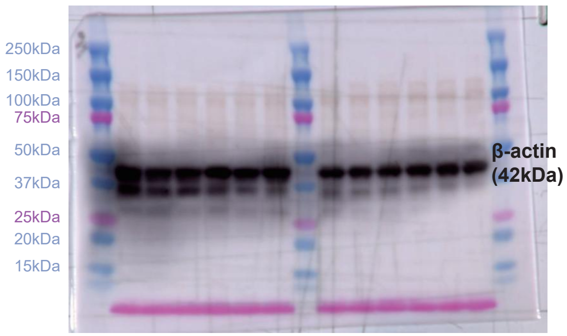


**Integrin αV expression on 4%-20% SDS-PAGE**

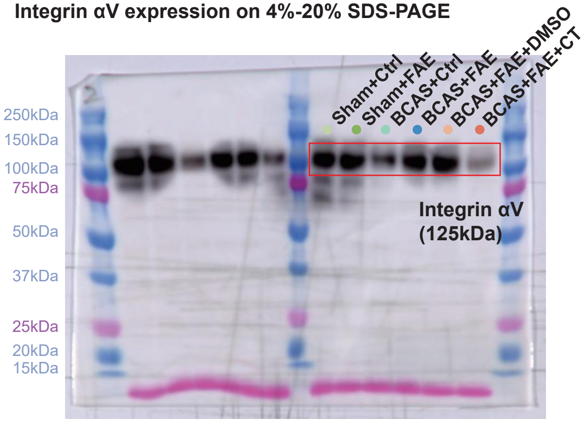


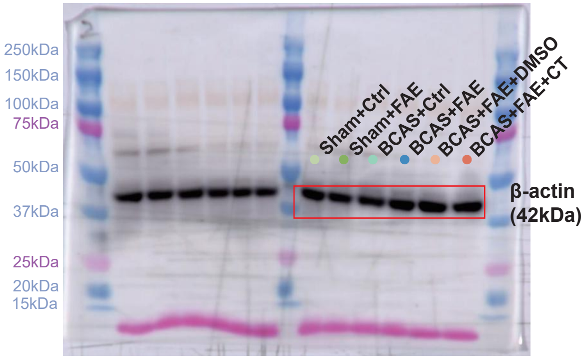


**Integrin β5 expression on 4%-20% SDS-PAGE**

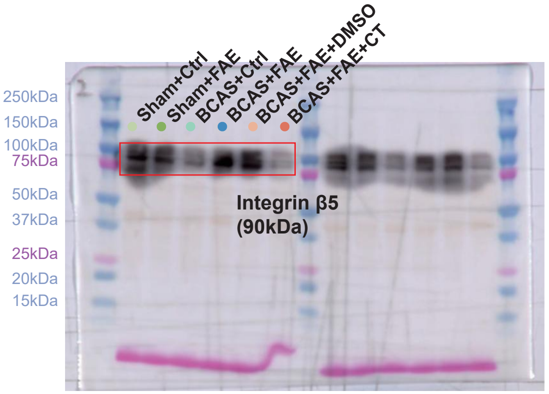


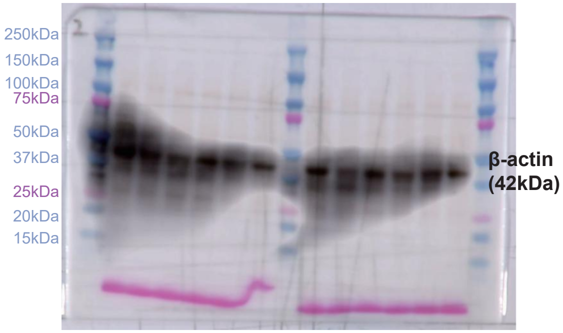

Full unedited gel for Fig. 7C

Caspase 3, Cleaved caspase 3 expression on 15% SDS-PAGE

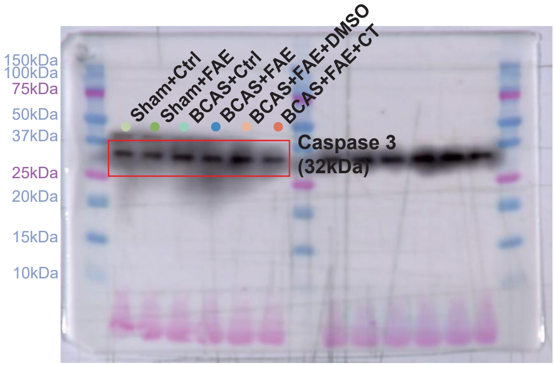


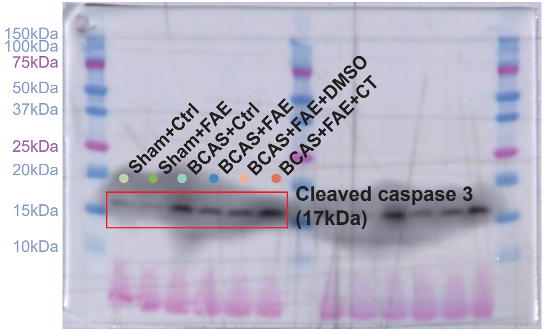


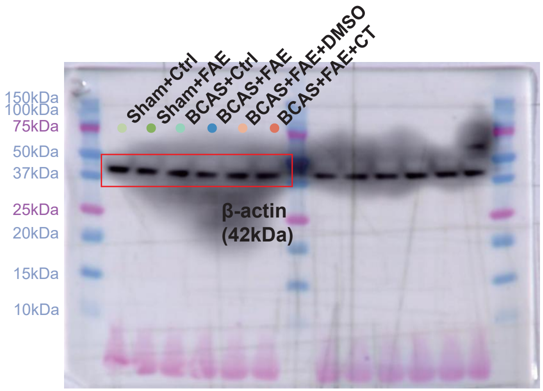

Full unedited gel for Fig. 7F

**Bcl2, Bax, Bcl-XL expression on 15% SDS-PAGE**

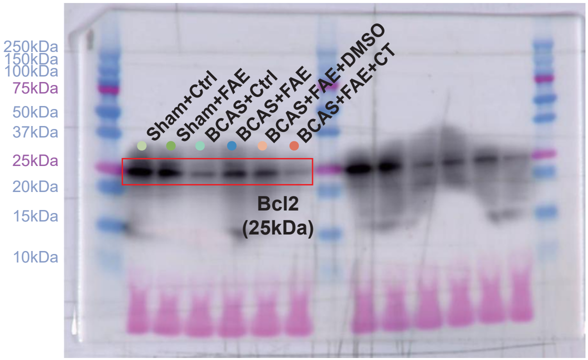


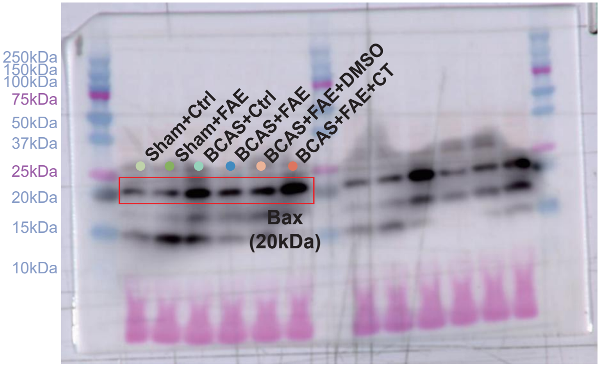


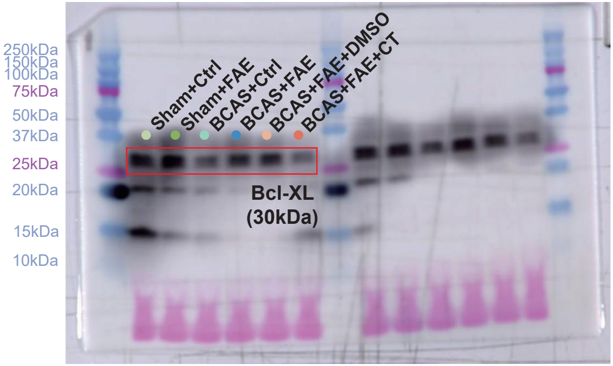


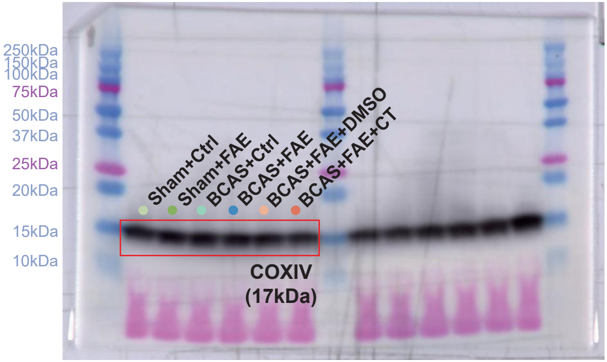

Full unedited gel for Fig. 8C

iNOS, CD86 expression in hippocampus on 4%-20% SDS-PAGE

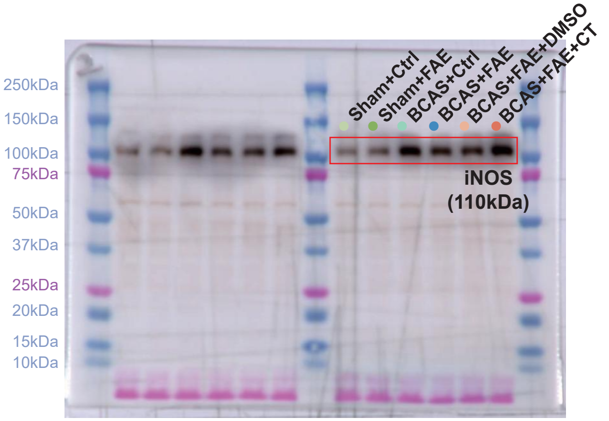


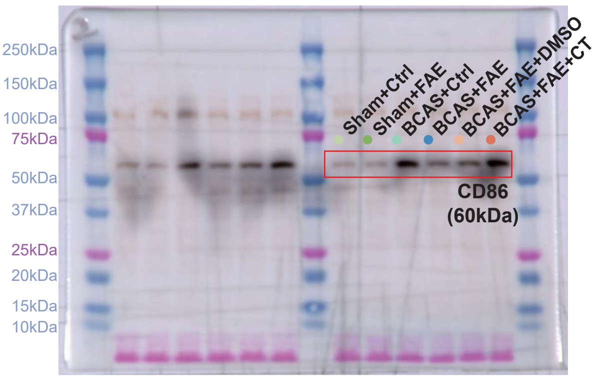


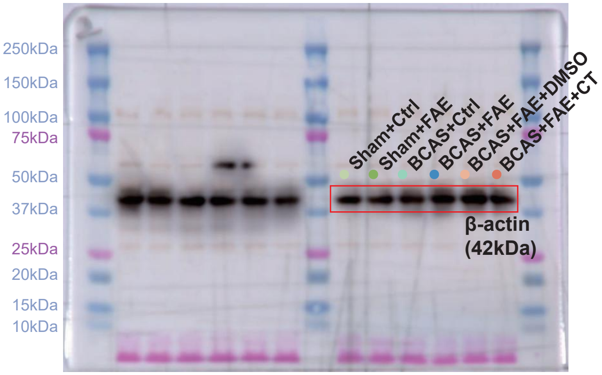


TNFα expression in hippocampus on 15% SDS-PAGE

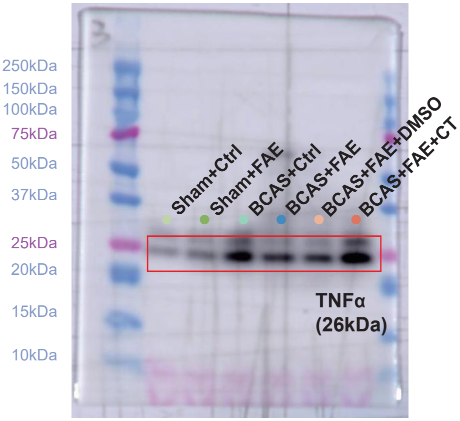


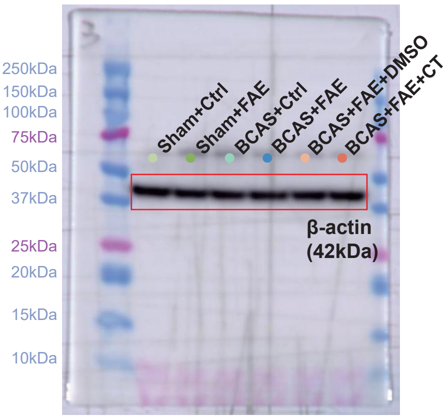

**Full unedited gel for Fig. 8G**

**iNOS, CD86 expression in cerebral cortex on 4%-20% SDS-PAGE**

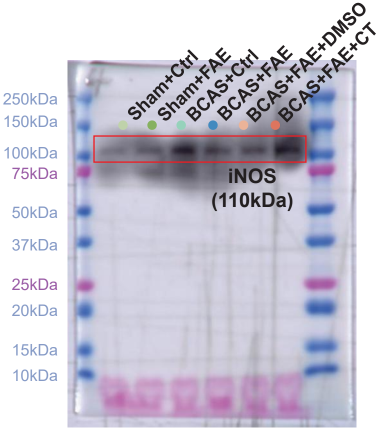


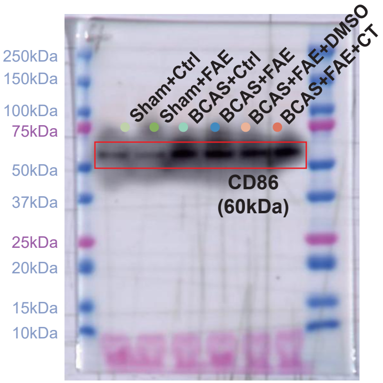


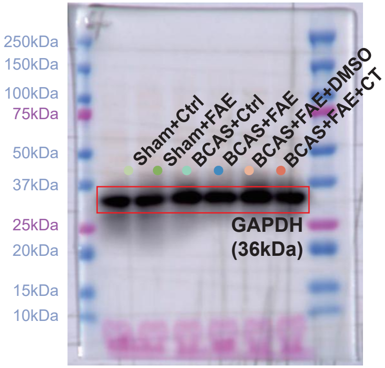


**TNFα expression in cerebral cortex on 15% SDS-PAGE**

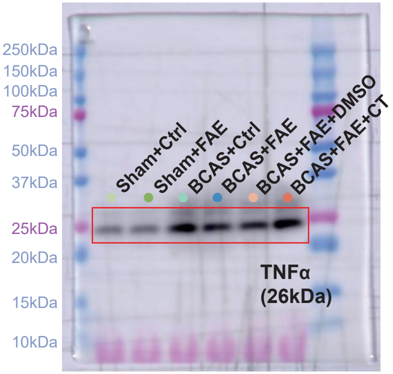


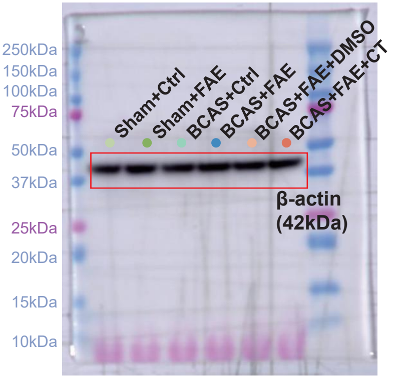

## Full unedited gel for Fig. 9

### Beclin 1 expression on 4%-20% SDS-PAGE

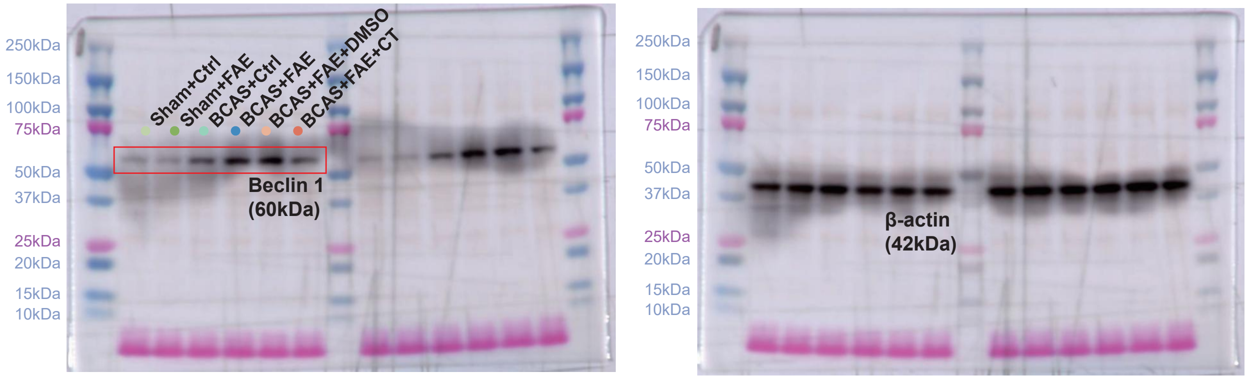


### SQSTM 1, LC3-I, LC3-II expression on 15% SDS-PAGE

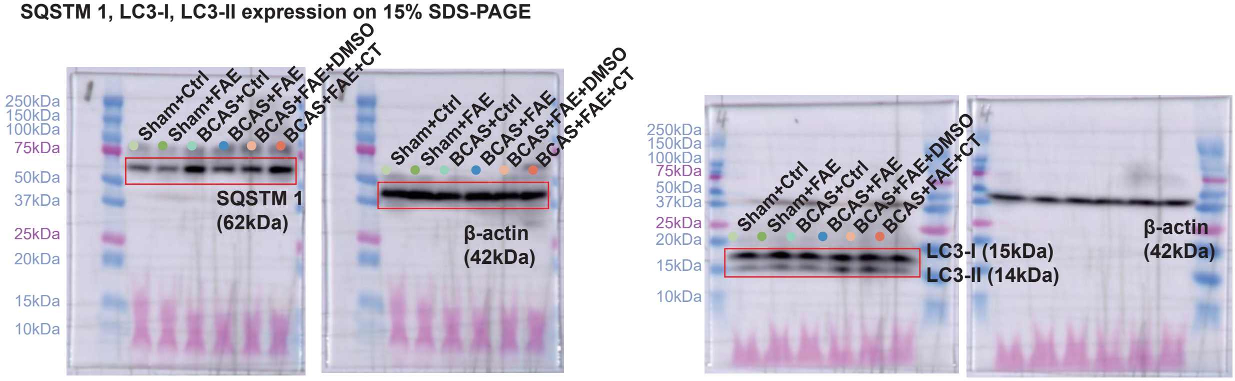


### pAMPK, AMPK expression on 4%-20% SDS-PAGE

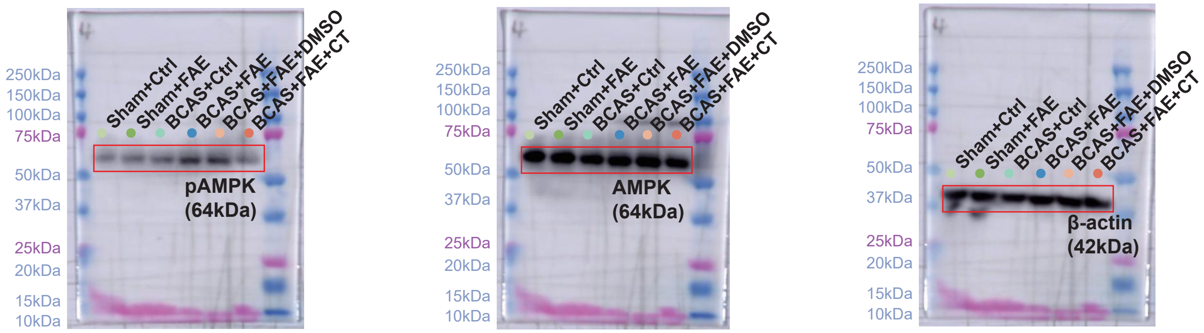


### pmTOR, mTOR expression on 4%-20% SDS-PAGE

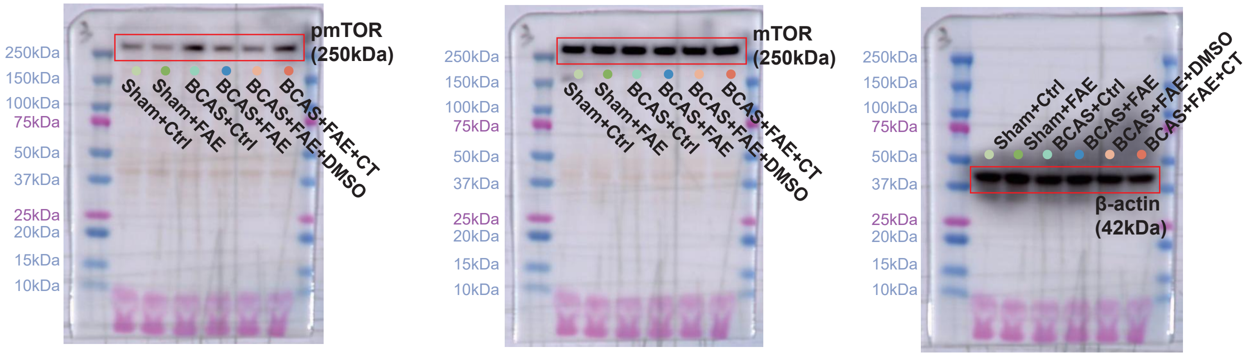

Full unedited gel for Additional file Fig. S2A

C3d expression on 4%-20% SDS-PAGE

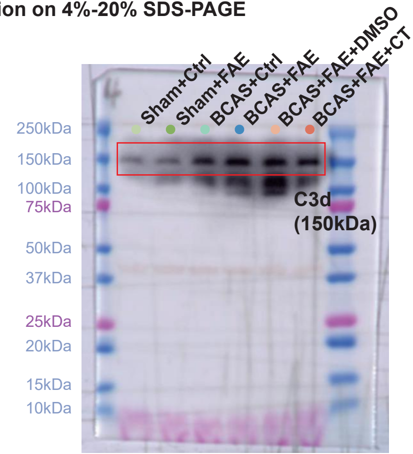


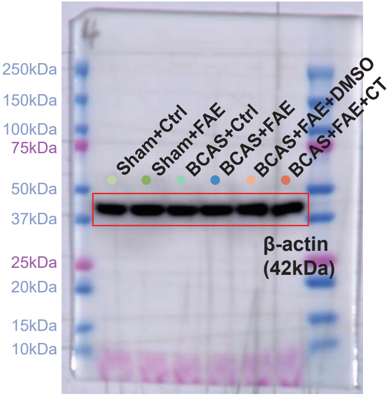

Supplement: Supplementary file 1 — Supplementary Material 1 [file 12974_2025_3493_MOESM1_ESM.pdf]
