# Supplementary material for: Exercise-induced irisin ameliorates cognitive impairment following chronic cerebral hypoperfusion by suppressing neuroinflammation and hippocampal neuronal apoptosis
Source: J Neuroinflammation. 2025 Jun 28;22:168. doi: 10.1186/s12974-025-03493-5 (PMC12205520; doi:10.1186/s12974-025-03493-5)
Supplement: Supplementary file 2 — Supplementary Material 2 [file 12974_2025_3493_MOESM2_ESM.pdf]

Table S1. Summary of experimental groups and mortality rate in Experiment 1.

Experimental 1	Rotarod test, ELISA, WB	IF	Mortality (%)	Subtotal
Sham	6	5	0	11
BCAS 1d	6	0	0	6
BCAS 3d	6	0	0	6
BCAS 7d	6	0	0	6
BCAS 14d	6	5	0	11
BCAS 21d	6	0	1	7
BCAS 28d	6	5	1	12
BCAS 35d	6	5	0	11
**Sum**	**48**	**20**	**2 (2.86)**	**70**

BCAS, bilateral common carotid artery stenosis. ELISA, enzyme-Linked immunosorbent assay. WB, western blotting. IF, Immunofluorescence staining.

Table S2. Summary of experimental groups and mortality rate in Experiment 2.1.

Experiment 2.1	ELISA, WB	IF	Mortality (%)	Subtotal
Sham+Ctrl	6	6	0	12
Sham+FAE	6	6	0	12
BCAS+Ctrl	6	6	1	13
BCAS+FAE	6	6	1	13
BCAS+FAE+DMSO	6	6	0	12
BCAS+FAE+CT	6	6	1	13
**Sum**	**36**	**36**	**3 (4.00)**	**75**

BCAS, bilateral common carotid artery stenosis. FAE, forced aerobic exercise. CT, cilengitide trifluoroacetate. ELISA, enzyme-Linked immunosorbent assay. WB, western blotting. IF, Immunofluorescence staining.

Table S3. Summary of experimental groups and mortality rate in Experiment 2.2.

Experiment 2.2	WB	IF	Mortality (%)	Subtotal
Sham+Ctrl	6	6	0	12
BCAS+Ctrl	6	6	0	12
Sham+FAE	6	6	0	12
BCAS+FAE	6	6	0	12
BCAS+FAE+DMSO	6	6	0	12
BCAS+FAE+CT	6	6	0	12
**Sum**	**36**	**36**	**0**	**72**

BCAS, bilateral common carotid artery stenosis. FAE, forced aerobic exercise. CT, cilengitide trifluoroacetate. WB, western blotting. IF, Immunofluorescence staining.

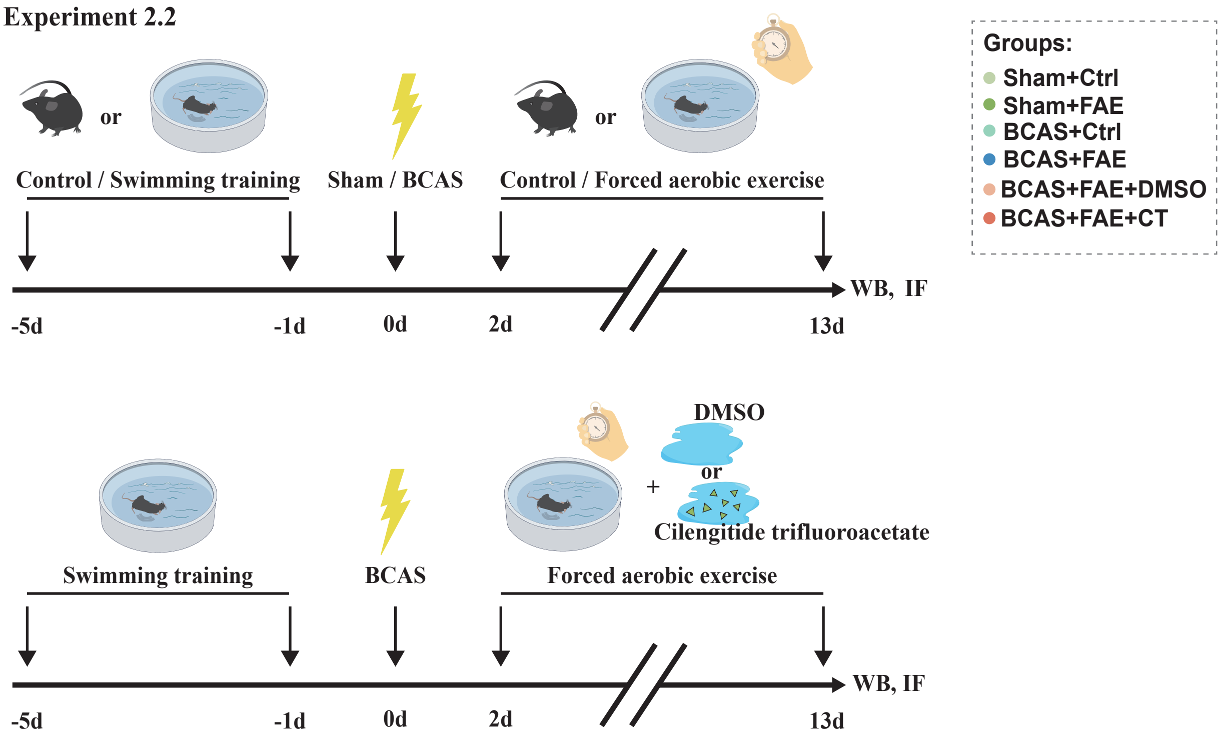

Fig. S1 Timeline of the Experiment 2.2.

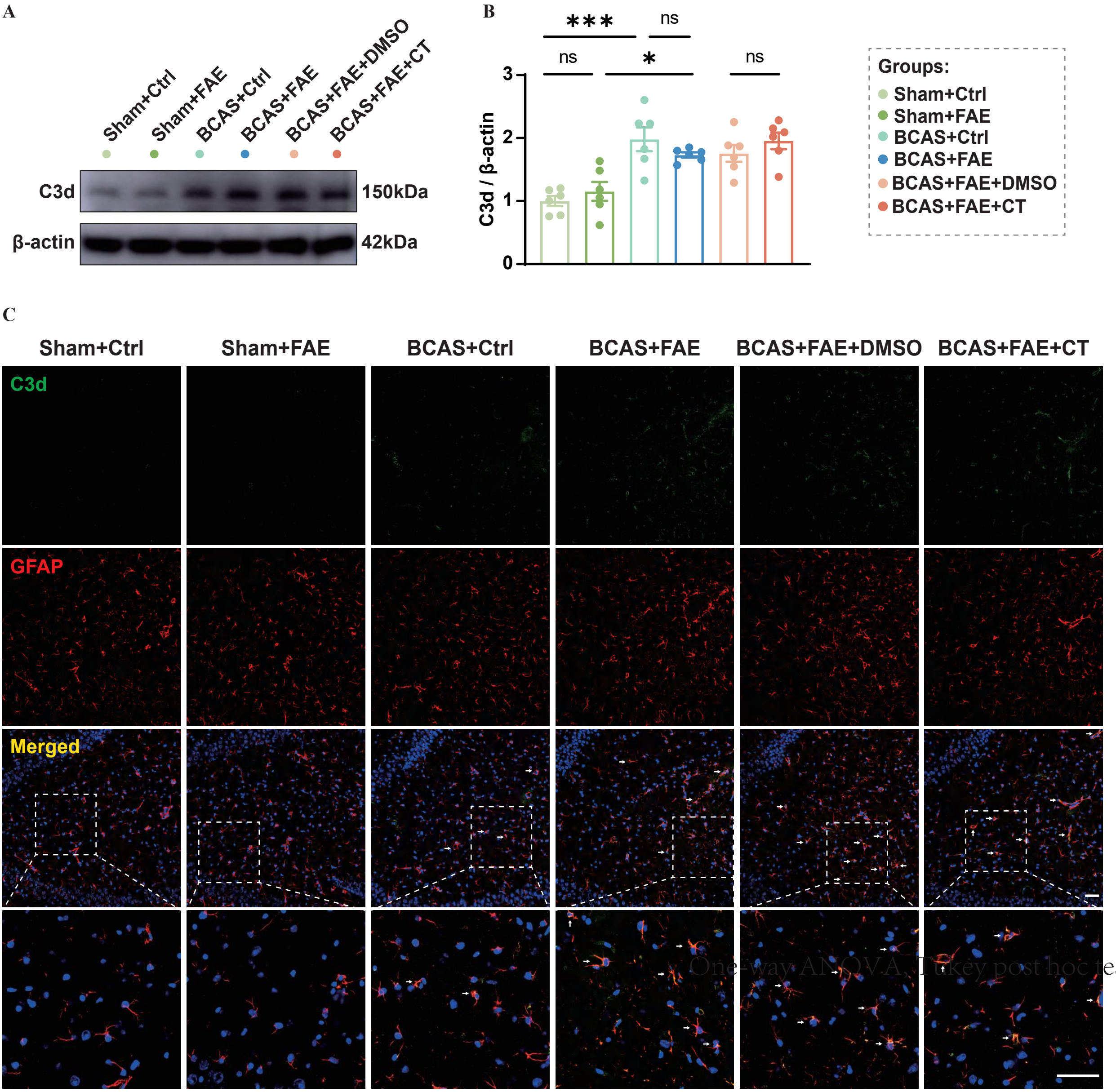

Fig. S2 Effects of increasing endogenous Irisin by FAE on astrocyte activation caused by CCH. **A, B** Western blot analysis of protein levels of C3d (pro-inflammatory markers for astrocytes) in brain after CCH, with quantification (band intensity normalized to β-actin) (n = 6/group). **C** Representative immunofluorescence images of pro-inflammatory astrocytes (GFAP+C3d+) in the hippocampus. Scale bar, 50 μm. One-way ANOVA, Tukey post hoc test (**B**). The data represent the mean ± SD, $p < 0.05$ was set as the threshold for significance. * $p < 0.05$, ** $p < 0.01$, *** $p < 0.001$ compared to the sham group.

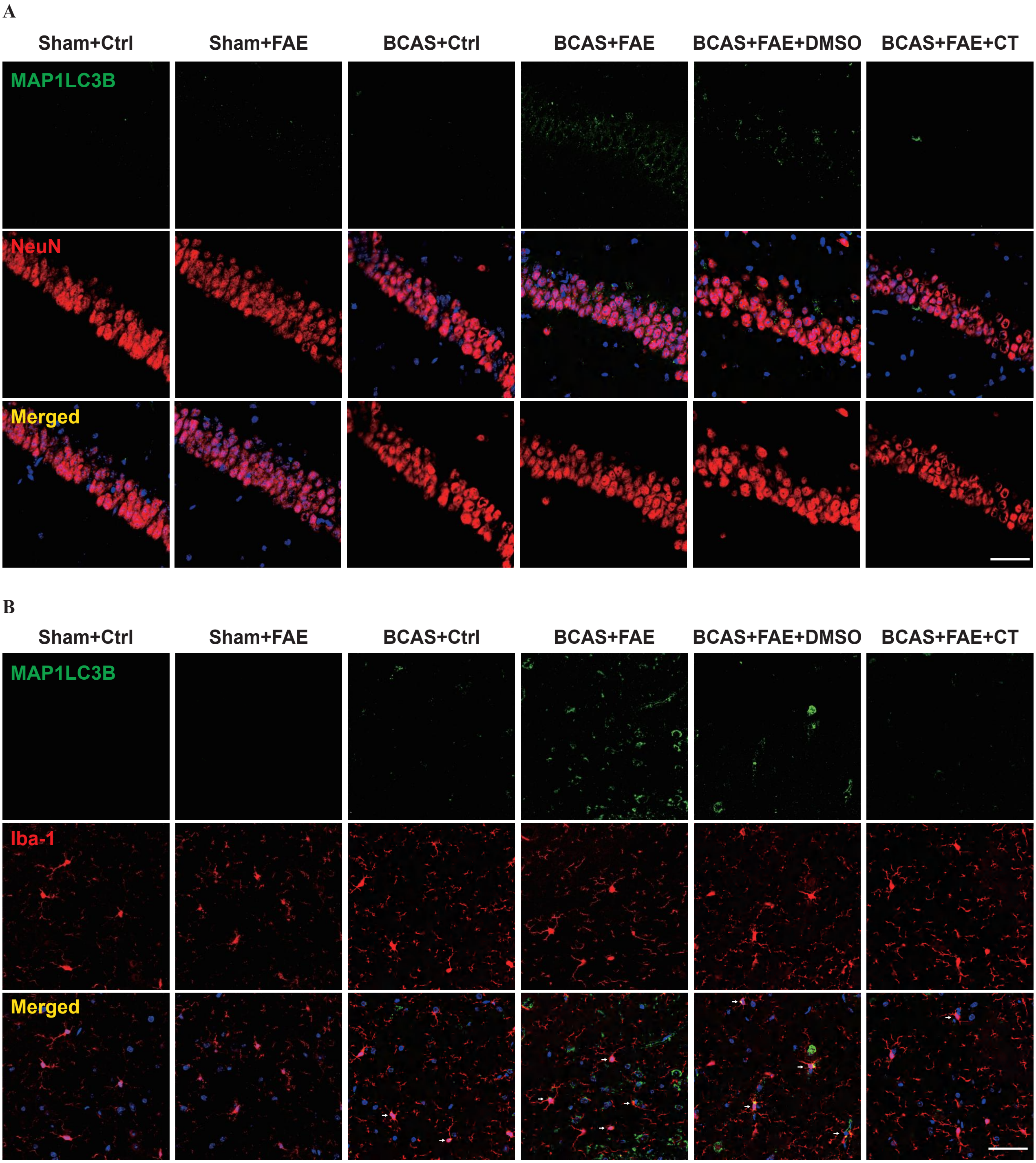

Fig. S2 Effects of increasing endogenous Irisin by FAE on neuronal and microglial autophagy. **A** Representative immunofluorescence images of MAP1LC3B+ hippocampal neurons. **B** Representative immunofluorescence images of MAP1LC3B+ astrocytes in the hippocampus. Scale bar, 50 μm.
